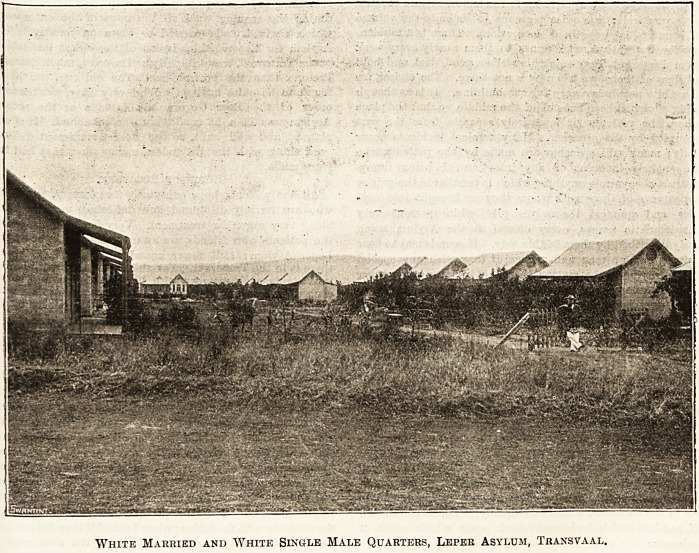# The Hospital. Nursing Section

**Published:** 1904-10-15

**Authors:** 


### The Hospital.

### Nursing Section.

Contributions for this Section of "The Hospital" should be addressed to the Editor, "The Hospital," Nursing Section, 28 & 29 Southampton Street, Strand, London, W.C.

No. 942.—Vol. XXXVII. *SATURDAY, OCTOBER* 15, 1904.

### Notes on News from the Nursing World.

### OUR CHRISTMAS DISTRIBUTION AND THE COMING DISTRESS.

In addition to the contributions to our Christmas distribution of articles of clothing from Miss Kate Saczkivic, No. 37 Royal National Pension Fund, and M. R., Ryde, we have to acknowledge this week the receipt of a large parcel from Miss Sharwood, Boyle Farm Cottage, Thames Ditton. According to all appearances there is likely to be an unusual amount of distress this winter among the very poor. As exceptional distress is sure to be accompanied by exceptional illness, the number of patients in the hospitals and infirmaries will probably be even greater than is generally the case in December. We hope, therefore, that we shall be able, by the kindness of our readers, to supply at Christmas more than the average quantity of warm clothing for the benefit of those who sorely need it. The extremity of the sick poor will doubtless prove the opportunity of the compassionate. All parcels should be addressed to the Editor, 28 and 29 Southampton Street Strand, London, W.C., with "Clothing Distribution" written upon the outside.

### NURSING AT KAISERSWERTH TO-DAY.

Every nurse, we are sure, will read with pleasure the account given in another page by a correspondent who visited Kaiserswerth a few weeks ago. As the institution where Florence Nightingale was trained, which has sent devoted nurses to all parts of the civilised world, it must always be a centre of interest. An illustration which accompanies the letterpress gives an idea of the quaint uniform, which will not be admired by some modern nurses. Neither will they approve the regulations with regard to the hair, the prohibition of haircurlers, and even of natural curly locks. At Kaiserswerth, however, the fashions change not; they are the same now as they were more than 60 years ago. Yet the nurses' quarters are comfortable, and, thanks to the benevolence of friends, they are furnished in quite an up-to-date style, while the hospital is lighted throughout by the electric light. Nothing could, in fact, be more striking than the contrast presented by the eagerness with which modern improvements for the benefit of the institution are adopted, and the old-world costumes and customs which our correspondent describes.

### THE CARE OF LEPERS IN THE TRANSVAAL.

In our issue of April 1902 we published a report of a visit to the Leper Asylum at Pretoria. To-day, from another pen, we give some quite different details which will be perused with special interest in the light of the recent appeal that has been made to the generosity of the British public. The appeal, we are glad to hear, met with a liberal response; and no one can fail to be impressed by the details supplied by our well-informed correspondent with the enormous value of the institution. The duties of the matron of the asylum are very varied, and, it will be seen that they even include acting as intermediary in a conflict between members of the native tribes. Those who have lately contributed to the fund for providing means for the recreation of the patients will judge from what our correspondent says how greatly their kindness will be appreciated by the poor folks who pathetically tell the matron of the asylum that she is "the only mother they have now."

### ABOLITION OF SCRUBBERS AT LIVERPOOL.

It will be observed from the report of our Commissioner to the David Lewis Northern Hospital at Liverpool, that under the auspices of the new matron, who was formerly assistant matron at Westminster Hospital, the daily scrubbers have been abolished, and resident wardmaids have taken their place. The change was made at the beginning of the year. The object was to give the probationers more time for their nursing duties by diminishing the amount of menial work; and the matron states that the experiment has been quite satisfactory all round. Since the engagement of the resident wardmaids, who sleep in the nurses' home, it has not been requisite for any of the day nursing staff to go on duty before 8 a.m.

### POST-GRADUATE LECTURES AT GUY'S.

The course of lectures inaugurated a year ago for the benefit of past nurses of Guy's Hospital proved so successful that the experiment is to be repeated this autumn, the lectures being given on Wednesday evenings, from 7.30 to 8.30, beginning on October 26th. The first lecture, on "First Aid," will be given by Miss Brereton, late sister at Guy's Hospital, and nursing sister, Army Nursing Service Reserve. This will be followed during November by lectures on "Tropical Diseases," by Miss M. B. Wood, sister, Government Hospital, Old Calabar, Southern Nigeria; on "District Nursing," by Miss E. M. Newton, lady superintendent, Nurses' Home, Ipswich; on "Mental Diseases," by Miss A. Oxley, matron, Bethel Hospital, Norwich; "Sanitation of Dwellings, etc.," by Mrs. Lyndon, late inspector of nuisances and sister of Guy's Hospital; and "Fevers and Infectious Diseases," by Miss M. Traill, matron, Royal Surrey County Hospital, Guildford. Further additions have been made to the course of post-graduate training, opportunities being now afforded for instruction in massage and Swedish drill, and suitable apparatus has been erected close to the swimming bath in the Nurses' Home, where lessons are given by a qualified sister. The use of electrical apparatus and baths is included in a course which takes six months for completion. For those entering now an examination will be held in February. Sisters and third-year nurses are eligible. The first lecture on Medical Nursing for first-year probationers was given on October 6th. The course lasts three months, and weekly classes are held at the close of each lecture, when instruction is given by the sisters.

### THE NEW REGULATIONS AT CHARING CROSS HOSPITAL.

The first examination of first year probationary nurses at Charing Cross Hospital, under the new regulations as to training, has just taken place. The questions were based on the elementary course, including anatomy and practical work, *e.g.* bed-making, application of splints, bandaging, etc., and a nurse failing to pass this examination would be referred back for six months, when it must be taken a second time. After passing this test, nurses are under continuous instruction by members of the medical and surgical staff, and the first lecture in the course on surgical nursing was given on Tuesday. This will be followed by a course on medical nursing, each course consisting of ten lectures. The whole of the ground covered will be reviewed at the end of the third year, and on the results of the examination then taken procedure to a fourth year as staff nurse will depend.

### A MATRON MARRIED IN A HOSPITAL PAVILION.

A wedding under exceptional conditions took place recently in Scotland. The bride was Miss Hempseed, matron of Coathill Hospital, Coatbridge, who was married to Mr. George Smillie. The wedding ceremony was performed in one of the pavilions of the hospital, which was opened that day for the first time, after being renovated and modernised. The whole of the hospital was hung with bunting and gaily decorated with flowers, and a number of invited guests were present. The bride's costume was of pale-green silk covered with crèpe-de-chine of a pretty champagne colour, the dress being much trimmed with insertion. The picture hat was *en suite*, and the bouquet of white carnations, sweet peas, and white heather. Her sister, Nurse Hempseed, and two little nieces were bridesmaids; they were attired in white silk and large white hats. Following the bridesmaids were Miss Hempseed's nurses, all of them dressed in the uniform of the hospital. A few days prior to the wedding the staff of the hospital entertained the matron, and presented her with a marble time-piece. The Hospital Committee, on behalf of the Town Council, under whom she served, also gave her a silver tea service and a purse of sovereigns.

### THE LONDON HOSPITAL AND SICK COOKERY.

At the London Hospital this term a course of 12 lectures on the general details of nursing will, as usual, be given by the matron. Sixteen lectures will be delivered on surgical nursing, and a similar number on medical nursing. Each course will be followed by an examination. There are special classes on bandaging and other subjects, and instruction and study classes are arranged for probationers preparing for the annual examination in July. In addition, systematic instruction in sick cookery is provided for by special classes, practical and theoretical examinations being held after each set of lessons by examiners from the National Training School of Cookery.

### ROYAL FREE HOSPITAL.

The lectures to nurses at the Royal Free Hospital have been arranged this year in two sets—*i.e.* for junior and senior nurses. From October to Christmas 12 lectures will be given on anatomy and physiology to first year nurses, and an examination will be held at the end of April and beginning of May in each year. Any nurse who fails will take the course again. Between Christmas and March of each year 14 lectures on medicine and surgery will be given to second and third year nurses, with an examination at the end of the third year, and this must be taken a second time in cases of failure to pass the first time. Three prizes will be given, consisting of books to the value of £3, to the three probationers who do best in the examination. Twenty nurses have joined the course now beginning, including four from the North-West London Hospital.

### NORWICH UNION NURSES' HOME.

The opening of the Nurses' Home at the Norwich Workhouse in Benthorpe Road, which took place the other day, marks another step forward in Poor-law nursing. The new building, which with the furniture has cost upwards of £2,250, has a handsome exterior on the city side of the workhouse, and the accommodation provided for the 14 members of the nursing staff in the interior appears to be both adequate and excellent. On the ground floor are an entrance hall, dining-room, sitting-room, kitchen, scullery and stores, five bedrooms and bath-room. A corridor runs from west to east of the building, and at the west end is a porch and entrance for general use, with an exit at the east end to the yard. On the first floor is the sitting-room of the superintendent nurse, and nine bedrooms, with bath-room. The rooms on the first floor all lead to a corridor, at the east end of which is an emergency door and a fire-escape staircase. Each floor has a large linen-closet, and in the corridors are ranges of cupboards with sliding doors. There is a clothes cupboard fitted in each bedroom. It is noteworthy that the erection of the home is entirely the outcome of the Guardians' own initiative, and is in no sense the outcome of pressure from the authorities at Whitehall.

### GLASGOW GENERAL NURSING ASSOCIATION.

At the annual meeting of the Glasgow General Nursing Association it was stated that 26 nurses were trained during the year and obtained certificates of merit, also that upwards of 200 maternity cases had been attended and nursed successfully. The financial position of the organisation, as shown in the report, is satisfactory, there being a balance of £28 in the hands of the treasurer. Having made such an excellent progress since its formation a year ago, the Association should have a very useful future before it.

### SERIOUS POSITION AT HASTINGS.

The position of the Borough of Hastings Nursing Association is, from a financial point of view, exceedingly serious. Representative members of the committee have carefully gone into the figures, comparing the items of income and expenditure with those of last year, and they estimate that by the end of the present year there will be a shortage of about £160. For several years there has been a credit balance, due to the surplus of original donations over and above the immediate cost for furniture and equipment. The credit balance of £62 in 1903 has this year been replaced by a debit of £40, or a loss of £100 on the eight months' working. As the committee affirm that this position is in no way due to want of economy, the conclusion is that the Hastings people must either be induced to give adequate support to the movement, or the number of nurses must be diminished and the sick poor suffer in consequence.

### CHELSEA HOSPITAL FOR WOMEN.

Autumn work has begun at Chelsea Hospital for Women, where members of the Junior Honorary Medical Staff in turn give courses of 12 lectures, followed by an examination on Anatomy and Physiology and Gynæcology. A course is also given by the assistant matron on "Nursing" and "Hospital Etiquette." These three courses follow one another with an interval of a few weeks between each, so that the subjects may all be taken during the first year. This arrangement is made in order to prevent nurses in their second year from being called away from the work of the wards. Any nurse failing to pass the first year examinations attends lectures during her second year with first year probationers.

### MASSAGE AT THE CITY OF LONDON CHEST HOSPITAL.

Instruction in massage has been added to the course of training for nurses at the City of London Hospital for Diseases of the Chest, lessons being given by the matron. The teaching is individual, and the entire course occupies three months, when an examination is held and a certificate given if proficient. Nurses in their second year, and preferably within six months of completing their training at the hospital, are eligible, and the introduction of the subject is proving a decided attraction to the course.

### AN AT-HOME AT ESSEX AND COLCHESTER HOSPITAL.

In the hope of attracting fresh subscribers to the Essex and Colchester Hospital the committee recently entertained about 700 of the townspeople, a number of leading residents being present. The guests were received by the members of the committee and the matron. In the out-patients' department the *x*-rays were explained, and in another part of the building the Finsen light and the electrical apparatus were manipulated by the house surgeon. The members of the fire brigade were in attendance with an up-to-date fire-escape, and there was quite a lively scene as sisters and nurses and many of the visitors, including the Mayor, descended in turn through the "shoot" from an upper window to the ground. The wards were inspected under the guidance of the sisters and nurses, and also the Nurses' Home.

### A DIFFICULTY AT EAST GRINSTEAD.

It was stated at the annual meeting of the East Grinstead Nursing Association that in May the question of permanently appointing the second nurse, who has lately been engaged temporarily, will be determined. The organisation has now been established for three years, and the fact that in the year just concluded the nurses paid 1,219 visits, proves that their services are appreciated. The subscriptions, however, though a little more than in the second year, were not up to those of the first. The Committee seem to think that if the nurses could get more cases from persons who can afford to pay fees, the financial position would improve. So it might in one direction, but the more the Association is supposed to depend upon the earnings of the nurses, the less the likelihood is of people regarding it as a charity entitled to their assistance.

### GUARDIANS AND DISTRICT ASSOCIATIONS.

At the last meeting of the Holbeach Guardians an application made by the Tydd St. Mary Nursing Association for a subscription was discussed. It was decided to refuse the request on the ground that persons receiving the services of the district nurse have to be subscribers to the association. At the same time it was determined to intimate to the association that if the committee are willing to adopt the course pursued by other similar organisations in the Holbeach Union, and allow the nurse to attend all cases of sickness where her services were required, an annual subscription will be voted. The condition is not unreasonable.

### THE NURSE'S MISTAKE.

In many towns the public offices, for the general convenience, are located under the same roof. That this occasionally leads to amusing incidents was shown in a suburb of Manchester the other day. A revision of the voters' list was taking place in one of the rooms of the building, and a nurse in uniform entered and walked up to the barrister's table. The barrister inquired for whom the nurse appeared. "For Mrs. ——," was the reply, mentioning the name of a lady residing in the district. "Why did she not come herself?" was the barrister's next question. The nurse, who was evidently surprised, replied quietly, "She could not very well." "What is it you want then?" "To register." "But," rejoined the barrister, "that I fear is impossible; there are no women on the parliamentary register. Do you not know that ladies as yet have no votes?" "Votes! votes!" exclaimed the nurse. "I don't want any votes. I want to register the birth of the child." Then, accompanied by a policeman, she was politely conducted to the office of the registrar of births, and this time she experienced no difficulty in accomplishing her business.

### SHORT ITEMS.

The dining-hall at Guy's Hospital has been recoloured a soft green, and Miss Chaplin's painting is now enclosed in a pretty oak beading.—The new home for the nurses at the Cancer Hospital, Fulham, will be quite finished by October 27th.—A nurse's home is to be erected by the Stepney Guardians in connection with their workhouse at Bromley, Middlesex, at a cost of £1,850, which includes certain alterations to the workhouse.—The first entertainment of the season for the in-patients of the Cancer Hospital, Brompton, was given last week by the Rev. L. J. Geffen, when an enjoyable programme was provided. Songs, 'cello solos, recitations, and piano and violin solos followed each other in quick succession and were received with enthusiasm.

### The Nursing Outlook.

"From magnanimity, all fear above;
From nobler recompense, above applause,
Which owes to man's short outlook all its charm."

### CERTIFICATES.

When the witnesses were being examined before the Parliamentary Committee on Registration there was a constant demand to see certificates, or to know the exact wording. In view of the present state of chaos it is interesting and instructive to have some knowledge of the various documents different nurses possess as proofs of their efficiency, and we have been at some trouble to collect specimens as varied as possible. First, here is the wording chosen by our largest hospital :—

LONDON HOSPITAL. Certificate of Training.

This is to certifiy that —— was received as a probationer on the —— day of —— and has completed her full term of two years' training in the medical and surgical wards of this hospital, both in day and night duty. During this time her work has been —— and her conduct has been ——.

Signed by Chairman, House Governor, and Matron.

Dated —— day of ——. — The name of —— has been entered in the London Hospital Register, and further particulars concerning her can be obtained at any time on application to the matron.

The virtue of this document is in the tail, for if all hospitals kept their register of nurses, and kept it up to date, many nursing scandals would be avoided.

Here are two provincial certificates :—

GENERAL HOSPITAL, TUNBRIDGE WELLS.

This is to certify that Miss —— resided at this hospital and received training as a probationary nurse from —— to ——, and that she has made satisfactory progress in practical and theoretical nursing, and that her conduct and attention to her duties have also been satisfactory.

Signed by Treasurer, House Surgeon and Matron.

ROYAL CORNWALL INFIRMARY, TRURO.
(Opened August 12th, 1799.)

This is to certify that —— entered the service of this infirmary as —— on —— and that she left the same on ——. Cause of leaving ——. General conduct ——.
Remarks ——.
Dated ——.
Signed by (Matron).
Approved by the Committee (Chairman's signature).

This is a Scotch specimen :—

GLASGOW ROYAL INFIRMARY. Training School for Nurses.

This certificate is awarded to —— for efficiency in medical and surgical nursing, as proved by work done in the wards over a period of —— years, and by written and oral examination.
Date ——.
Signed (Superintendent and Matron).
This is on parchment.

The worst example before us is this, but it is dated ten years ago, and perhaps is not in use now :—

THE ROYAL HANTS COUNTY HOSPITAL.

This is to certify that —— has attended the annual course of lectures delivered to the lady probationers and nurses of this hospital.
Signed ——, Lecturer.

As regards monthly nursing the following is the wording of the Queen Charlotte's Hospital, printed on parchment :—

No. ——.

QUEEN CHARLOTTE'S LYING-IN HOSPITAL.
(Patron: Her Majesty the Queen. Founded 1752. Incorporated by Royal Charter 1885.)

These are to certify that ——, of ——, has been a pupil of this hospital; that she has received instruction in the duties of a monthly nurse, and has had special instruction for an additional period; and that the said —— has shown herself competent to discharge the duties of the position.
Dated this —— day of ——.
Signed by two Physicians, Matron, and Secretary.

One of the most truly Irish and bewildering documents is the following—we take it to be a midwifery certificate :—

This certificate does not confer any legal right to admission to the Medical Register.

ROTUNDA LYING-IN HOSPITAL, DUBLIN.

We, the Master and Assistants of the Hospital for the Relief of Poor Lying-in Women in Dublin, do hereby certify that Nurse —— has regularly attended in this hospital during the space of —— months, the course of practical and oral instruction for nursetenders (or midwives) as provided in the Charter, and that she has passed an examination before us. In testimony whereof we hereunto annex our names and seal of office.
—— day of ——.
(Signed by Master, two Assistants, and the Secretary).

Now for a specimen from an infirmary :—

SHEFFIELD UNION WORKHOUSE INFIRMARY.

This is to certify that —— has satisfactorily completed a course of three years' training as a hospital nurse, and has diligently attended the lectures and demonstrations on the Principles and Practice of Nursing, at this Infirmary, from —— to ——. That she has satisfactorily passed an examination in the subjects treated upon therein, and is considered to be entitled to a Certificate of Efficiency. The estimate of her character and qualifications are as follows, viz.:—Conduct ——. Medical Cases ——. Surgical ——. Midwifery ——.

Signed by Visiting Physician, Honorary Examiner, and Infirmary Superintendent.

This Certificate was presented at a meeting of the Committee of the Infirmary held on —— by —— Chairman of the Committee.

This is the most elaborate specimen quoted, so far: the blanks on duties are filled by Excellent, Very Good, or Efficient, according to work done.

A splendidly designed certificate on parchment for mental nurses runs as follows :—

NORTHAMPTON COUNTY LUNATIC ASYLUM.

This is to certify that —— has satisfactorily completed three years' training in the wards of this asylum; she has received instructions in the following subjects, and has passed examinations, practical and theoretical, in them :—First Aid to the Injured, Duties in the Sick Room, Care of the Insane. She is competent to take charge of Mental Cases.

Signed by two Medical Officers, Chairman, and one Member of Committee.

Of the handsome certificate of the Royal National Pension Fund, of the certificates of honour given in some hospitals to those who pass well in the examinations, of the Hygiene, Cookery, and other added certificates, we have no room to write at present; but certainly, from a study of some two dozen certificates now before us, we are convinced that one of the first duties of the Nursing Council should be to see that all certificates are dated, are plainly worded, are fully filled in, and are brought more into harmony with one another.

### Lectures upon the Nursing of Infectious Diseases.

By F. J. Woollacott, M.A., M.D., B.S.Oxon, D.P.H., Senior Assistant Medical Officer, Park Hospital, Metropolitan Asylums Board, Hither Green.

### LECTURE VII.—DIPHTHERIA.

The name Diphtheria is derived from a Greek word meaning a membrane, and, as we shall see, its most characteristic symptom is the appearance of a membranous deposit in the throat or elsewhere. It is a highly infectious disease, and like scarlet fever is most prevalent and most dangerous among children, although adults are by no means exempt. As a rule infection spreads from person to person, either directly or through the agency of clothes. Many outbreaks, however, have been traced to the consumption of milk containing the specific virus, and it is highly probable that some domestic animals, such as cats, occasionally suffer from the disease and transmit it to human beings. Like scarlet fever it is most active during the last quarter of the year, and sinks to a minimum in the spring and early summer. It is an interesting fact that weather conditions have a marked influence on its prevalence. After a period of scanty rainfall the number of cases begins to increase, and two or three dry years are nearly always followed by a severe outbreak. On the other hand, continuous and heavy rain acts as a check on the spread of the disease. This has been well illustrated recently. The past two years have been very wet in this country, and, although in the dry years immediately preceding, diphtheria had been very prevalent and of a severe type, it has since undergone a considerable diminution in both respects.

The incubation period is short, as a rule from two to four days, and always under a week.

The onset may be acute, but often it is insidious and easily overlooked. The patient seems languid and out of sorts, and perhaps a little feverish, and may exhibit the symptoms of a slight cold in the head. After a day or two the glands of the neck are seen to be enlarged, and then attention is drawn to the state of the throat. In consequence of the absence of pain, it frequently happens that the disease has made considerable progress before its presence is suspected, especially in young children unable to speak; so that it has become a general practice among medical men to examine the throat in any illness where the symptoms are vague and doubtful. In more acute attacks the signs of sore throat may be prominent from the first, often accompanied by vomiting and headache. Occasionally the earliest manifestation is a croupy cough, showing that the larynx is involved.

If we have the opportunity of inspecting the throat when the disease is beginning, the following is what we see. The tonsils are a little swollen and reddened, and on their surface are little whitish dots, streaks, or patches. These rapidly increase in size, until in a day or two, or even less, the whole of the tonsils may be covered by a continuous sheet of exudation, which soon spreads on to the adjacent palate. This exudation is known as membrane. At first thin and whitish, it soon grows thick and tough, and becomes yellowish or of a dirty grey colour. If we were to tear away a piece with forceps the surface beneath would be found raw and bleeding, and the membrane would soon reform. Unless the disease be checked by appropriate treatment, the exudation will, in a severe case, spread rapidly in all directions. The whole of the soft palate will be covered, together with the walls of the pharynx. The nasal cavities will be similarly attacked, and the process may even extend along the Eustachian tubes to the ear and set up ear discharge, or may invade the windpipe and lungs and give rise to croup and pneumonia. At the same time, the glands of the neck become swollen and painful, and occasionally an abscess forms. The temperature in the early stages is usually raised a few degrees; but, as a rule, it soon falls to the normal, or even lower. Whether high or low, it furnishes no indication as to the extent of the disease.

A severe and neglected case of diphtheria presents a very distressing spectacle. The patient, usually a young child, is in a condition of intense discomfort. The throat is so clogged up by the mass of membrane that swallowing is rendered painful and difficult, and speech indistinct. The nose is blocked so that breathing is more or less obstructed, and from the nostrils a thin, often blood-stained, discharge constantly oozes. From both nose and throat a horrible and very characteristic fœtor is given off. The neck is greatly swollen, and the skin covering it often reddened. The patient is pale and restless; sleep is almost impossible, and he rapidly becomes weak and exhausted. These are the appearances in very bad cases, which fortunately form only a small minority of the whole. In milder cases, and in those treated in good time, so that the progress of the disease is checked, although the same symptoms are present, they are much less marked. There is less formation of membrane, less swelling of the neck, and less nasal obstruction, while the foul smell is less noticeable or altogether absent. The general condition, too, of the patient is more satisfactory, for he is able to take food without pain and to breathe without discomfort. Frequently the attack is milder still, and with appropriate and early treatment there may be no other symptoms than a slight sore throat, and perhaps a little nasal discharge. After the disease has lasted a few days albuminuria frequently makes its appearance. In mild cases it is usually slight, and persists for a short time only; but in severer cases it may be plentiful and long continued. The quantity of albumen may indeed be taken as a useful guide to the severity of the disease, for, as a general rule, the more albumen the more grave is the attack. The amount of urine is at times much diminished, so that only 2 oz. or 3 oz. are passed in the 24 hours. Occasionally total suppression occurs. This is always a sign of very great danger.

A severe case of diphtheria sometimes assumes what is known as the hæmorrhagic type, that is to say, the patient shows a tendency to bleed from the nose and throat, and into the skin and internal organs. The discharges from the two former are mixed with blood, which constantly oozes from the mucous membrane; there is blood in the vomit, and the stools are black. The skin shows hæmorrhages of two varieties—(1) Petechiæ, like flea-bites; (2) small blue spots, like bruises. The latter usually occur over bony prominences, and are due to slight pressure which in the healthy state would have no ill effect. To these blue spots the very greatest importance attaches, for, whether few or many, they indicate that the condition of the patient is hopeless. They are more than a danger-signal; they are a death-warrant, as in these cases recovery is practically unknown. They indicate that a very large amount of the poison of the disease has been absorbed into the system.

Under favourable circumstances, after a variable time depending largely on the treatment adopted, the local symptoms begin to subside. The membrane separates, the swelling of the neck goes down, the nasal discharge ceases, and the patient becomes comfortable, able to sleep well and to take food. The constitutional symptoms, however, persist much later. Anæmia and debility, especially after a severe attack, are often long continued, and complications, chief of which is paralysis, may appear and sometimes prove fatal. The disease shows a special tendency to weaken and depress the heart, and even after a comparatively mild attack its action and rhythm may be disturbed. The pulse may be very much slowed, though more often its rate is increased. It is usually more or less irregular, and occasionally intermittent. From time to time it varies considerably, and any exertion or excitement may alter it to a very marked degree. This condition may last for several weeks before complete recovery takes place, and during the whole of the time the case must be carefully watched. Under less favourable circumstances the patient does not survive the early stages of the disease. Death may result from extension of the membrane to the larynx and lungs, but more commonly from heart failure, or cardiac paralysis as it is frequently called. Besides weakness and altered rhythm of the pulse, the failure of the heart manifests itself by the following signs:—The patient becomes restless, or at times drowsy, the extremities and the surface generally grow cold, and the pallor of the face increases. Vomiting, too, is rarely absent, and is usually so troublesome that no food can be retained. In addition there is frequently a sense of discomfort or pain in the abdomen or over the heart, which at times may be very severe. Any one of these symptoms is a signal of danger, and when the whole group is present there is practically no hope left. The patient may linger for hours or even days but sooner or later succumbs, and in young children the fatal termination is often preceded by convulsions. Sudden death is very rarely seen if proper treatment be adopted. In many cases the first warning is given by the onset of vomiting, a symptom which should never, under any circumstances, be lightly regarded, in the whole course of diphtheria. It should always be reported at once to the doctor.

Though the liability to heart failure is greatest in the acute stage, *i.e.* before the local symptoms have subsided and immediately after, yet it is sometimes seen later in the disease, often in association with various forms of paralysis. The symptoms, of which vomiting is prominent, are similar to those just described, but on the whole of a less severe type. There is always grave danger, but recovery frequently occurs if the case be properly managed.

### District Nursing in the South Sea Islands.

It must not be supposed that district nursing in the South Seas corresponds exactly with what is understood by that term in England. I did not go out to the South Pacific with the primary intention of doing district work, but the need for a nurse arose, or rather existed, and as far as strength and leisure permitted, I bent my shoulders to the burden.

### My First Case.

My first case was a cancer patient—the remains of what had been a fine old Scotch settler. Years before I saw him he ought to have submitted to an operation, but he persistently turned a deaf ear to all the doctor's exhortations. At last, agony and a desire to prolong life if only a little in order to see his daughter married, induced the old Scot to yield. An old native-made stone house, once a mission dispensary, was our "theatre," an ordinary bed our operating-table, the surgeons and anæsthetist were the German doctor resident on the island and his *confrères* from the two German warships then in port, whilst the pulse-teller was a very clever all-round "handy man" holding at that time the official position of head of police. It is astonishing, until experience has made you take things almost for granted, what men and women can turn their hands to when thrown upon their own resources, and any way, it needs a certain amount of grit to become a settler.

My own position was certainly novel. I was ordered to sit on the man's legs, and keep him perfectly quiet if I could. Doctors on South Sea Islands are not in a position to be waited on hand and foot by trained nurses, and so my dear old doctor had grown accustomed to get his own instruments ready and wait upon himself. I took up my position obediently, and with intense interest watched the manipulations of the surgeons. They were removing the eye, and as I watched my one conscious thought seemed to be when will they be able to get to the end. At last they desisted; the cancerous growth had penetrated much too far for any hope of recovery ever to have been entertained, but the agony was lessened and life prolonged sufficiently to admit of the realisation of the old man's wish. It was just at this juncture that a scheme, originated by H.B.M. Consul—viz., the establishment of a Benevolent Society for Sick Settlers, had been successfully floated; so before long I was able to superintend the removal of this patient to the cottage temporarily hired by the society. Here the Scotsman lingered for a time, growing daily thinner and thinner, and at last he "crossed the bar." During my work among the sick on that island I had abundant opportunity of proving how tender and helpful some men can be in times of sickness. With one exception all my patients were men, and all my nursing assistants also.

### My Principal Assistant.

There was one fine "young merchant" (save for his tendency to drink) whom in my mind I sometimes dubbed "my scout," the first intelligence of my help being needed seemed so often to come through him, and his waggon, or buggy, according to requirement, was always at my disposal. I remember one Saturday he suddenly appeared on my verandah, exclaiming almost at once, "Oh, old M—— is very ill! can you come?" "Yes, I'll come; do you want me at once?" "Yes, straight away." I slipped on my hat, took up my umbrella, and as we walked along the "Beach" he told me where to find the old settler. And what a find! Ah! me; some of the saddest sights in life are the closing scenes in the lives of men, who but for drink might have been so different. And yet associated with all this sadness is a joke that clings still. The room had to be aired, it was foul; the patient had to be washed, he was dirty. I knew the doctor had given him up, but the last hours had to be lived through, and air and cleanliness had to be obtained. My instructions were carried out by my handy assistant, and in comparative comfort the wasted life came to an end. After the patient's death, I happened to meet some Government officials well known to me; they paused for a few moments' chat, during which one said, "So old M—— is dead!" "Yes." "Well, you see, you would have him washed, and we are told he had not washed for six months, so it was your bath that killed him." Meanwhile, better situated premises for the work of the Society had been obtained, a wooden structure erected on an open space facing the Pacific, and enjoying the coolest breeze obtainable in that island. It was not a large building, only able to accommodate three patients at a time, but it answered our requirements then. Again, one Saturday my "scout" appeared to ask if I had heard that

### DISTRICT NURSING IN THE SOUTH SEA ISLANDS—*Continued.*

H—— was very ill. I had not. Something of this H—— I knew, for his mother in the Old Country had a short time back written to me to ask if I would try to bring about a reconciliation between H—— and his brother, both of them traders on this South Sea Island.

### A Victim of Disobedience.

Hitherto my efforts had proved futile, and now, H—— was very ill—a relapse in "dengue" fever, brought on by food taken contrary to the doctor's orders. It was not quite so easy to take over this case as it had been with old M——, there was a little more formality to go through. First I had to get the Consul's permission to use the Benevolent Cottage—for the Consul was the president of our Benevolent Society. Then the doctor's order for removal had to be obtained, and lastly the transportation of the patient had to be cared for. However, my "scout" seemed to consider that everything would be satisfactorily arranged, for his last words to me as I mounted my horse to gallop off to the Consul's were, "I'll have the waggon prepared, and see that A—— (my male assistant) is ready." The Consul readily gave the permission sought for, adding, "You ride back to Dr. F—— and get his signed permit of removal, and I'll walk on to H——'s house and await you there, for I'll have to affix the seal on his papers before he leaves home." I quickly turned my horse's head, gave him the rein and soon arrived at Dr. F——'s. He, too, granted what I sought, saying, "Get him away to Vaiala (the Benevolent Grounds) as soon as you can, it is his only chance—if it fully answers, the change for the better will take place in about seven days—any way, if he die, it will be a nicer place to die in." Armed with the signed permit I quickly rode to H——'s house, witnessed the affixing of the seals, and then hurried off to the "scout" and handed into his charge the getting of the patient into the waggon, etc., while I hastened on to our cottage to see the bed and other necessary things were in order. Two other settlers volunteered assistance in the nursing, and everything that could be done for H—— was done, but he fell a victim to disobedience to doctor's orders—not his disobedience, but that of his native wife.

### A Little Diphtheria Patient.

One morning, very early, a little tap on the French window of my bedroom wakened me with a start. "Who is there?" "It is I," came back the answer in German, and I recognised the voice of a young German girl well known to me. "What do you want?" "K—— (naming a younger sister) is very ill; she is choking." I sprang out of bed, slipped on my clothes, and hastened on to the verandah, where to my astonishment I found Madame C——, the child's mother. Restless anxiety had rendered it impossible for her to remain at home, so, leaving the child propped up and the husband preparing a fire to procure hot water, she had come hoping to get help from me. Fortunately, one of the doctors of a British man-of-war was sleeping ashore that night in the compound where my home was. A step or two brought me to his abode, a smart rap on the door awakened him. My voice was recognised, and having told him quickly what I knew and what I surmised, he asked me to go on at once, begin treatment, and he would dress and follow on as soon as possible. This he did. As I feared, the child was ill with diphtheria. Our promptitude happily averted any disaster, the mother's fears were calmed, instructions given, quarantine enforced, and after a month the children were able to resume school work, no second case occurring in the family. I have still in my possession the handsome lamp and brooch which testify to the gratitude felt for the help rendered. The last time my services were requisitioned in this kind of way was shortly before my departure from this island. I was taking my constitutional one afternoon when I heard my name called. I turned and saw the wife of a settler beckoning me. I retraced my steps and was greeted with these rather peculiar remarks:—"Would you like some real butter? Come in and have some tea. I have had a gift from the man-of-war." (*En parenthèse* be it remarked, in tropical lands butter often becomes liquid fat. "Real" butter in this case meant a portion of that edible straight off the ice from the man-of-war refrigerator.) Over the teacups I learned that the Consul was seriously ill, that my hostess expected the doctor from the warship to go by any moment, and I was asked did I think I could help in any way, for if I could she would call the doctor in, as she knew he wanted the Consul moved to a healthier and more cheerful spot. A sad bereavement had befallen this patient not very long before, the loved one's remains had been buried near the home, and this constant reminder of his loss, coupled with an attack of the dengue fever, had stricken our Consul very low. Yes, I thought I could arrange for him and his young daughter with her maid to be received in the Mission. Just then the doctor passed, and my hostess at once called him in. A few words settled matters, and I started for the Consulate with the M.D. Sickness as well as sorrow levels all distinctions, and I think that the Consul was really glad to obey orders, even those of a nurse. A few preliminaries had to be arranged, so I hurried back to the Mission compound to settle these, and then prepared to return to the Consulate to superintend the removal of the patients, for the little girl was ill too.

### Removing the Consul in a Storm.

One needs to live in the tropics fully to understand how quickly a storm can rise. As I left the mission-house I saw ominous signs in the sky, the doctor had seen them too, and as I hurried across the compound his voice from the verandah of a hotel close by, informed me he had to get back to his ship and duty, and that he was going at once because of the changing weather, so he must leave the responsibility entirely with me. Being a quick walker, I could, he thought, outrace the storm, but I would have to go ahead. This I did. During my absence the consular boat had been prepared and the native boatmen were awaiting orders. The Consul had to come by way of the lagoon—no one would venture out to take out buggy or horses in the teeth of what threatened to be a severe blow, but the boatmen knew their work, and pulled away with a will. Having seen the Consul safely embarked under the care of the child's maid, I returned to fulfil my share of the pilotage, viz. the carrying of the little girl to brighter quarters. I had not realised before how very heavily a sick child of some summers can lie on even willing arms. And then the race with the storm began in grim earnest. Through the lagoon splashed the oars, along the "Beach" road hastened I, while overhead the clouds gathered and darkened, and in the distance muttered the thunder and gleamed the lightning. All this mattered in a way but little, if only no rain would fall till my charges were safely housed. The child and I got in first, and oh, how thankful I was to put her safely in the bed prepared, and leave her with the missionary's wife! Then the missionary and I hastened to the landing-stage to receive the Consul; he was well wrapped up in blankets, and best of all, only just the first big drop or so of the coming torrents fell on him as he slowly mounted the verandah steps. They both got well and strong, and before I sailed for another island, I had the joy of wishing them *bon voyage*, as they started for dear old England.

### The Nurses of the David Lewis Northern Hospital, Liverpool.

INTERVIEW WITH THE MATRON. BY OUR COMMISSIONER.

Of no modern institution are the citizens of Liverpool more justly proud than the splendid hospital which was opened in March 1903, thanks to the generosity of the David Lewis trustees. Six months later Miss Anderson, who had been matron of the original institution, resigned, and her place was taken by Miss A. C. Glover, who, having been trained at the London Hospital, was for five years assistant matron of Westminster Hospital. After a year's experience as head of the training school at the David Lewis Northern Hospital, she has grown accustomed to the somewhat different conditions of work in Liverpool, which, however, she finds quite pleasant and encouraging. If the Westminster Hospital is only a short distance from the Thames, the David Lewis Northern Hospital overlooks the Mersey, and this, as I realised when I accompanied the matron through a number of the wards is a great advantage to both patients and nurses. There is, of course, the greater advantage that the hospital itself is built on the most modern lines, and not only contains every possible convenience but is also equipped with the very latest scientific appliances and sanitary improvements.

"And how is the nursing carried on under such favourable auspices as I have observed this afternoon?" was the first question I addressed to Miss Glover when we returned to her tastefully-appointed sitting-room.

"I had better begin by telling you about the staff," replied the matron. "There are sixty sisters, staff nurses, and probationers in the hospital, and a private staff of fourteen so far. We hope that the private staff is growing. The number of sisters is twelve, including the assistant matron (who acts as home-sister), the night sister, the Light department sister, and the housekeeper sister."

### Housekeeping Experience.

"I attach so much importance to housekeeping experience that a nurse in her third year is assigned the duties of assistant housekeeper-nurse for a period of three months."

"How many of the probationers are in their first year?"

"Twenty-six. The remainder are in their second and third years. The training is for four years, three in the hospital and one on the private staff. I think that the four years' training suits both the hospital and the nurses. No objection has ever, that I am aware of, been made to the extra year."

No. 1 Ward, David Lewis Northern Hospital, Liverpool.

### Four Years' Training.

"What is the age of admission?"

"Twenty-three. Each nurse comes on trial for six months, and if she remains the whole of that time counts as a portion of the four years. A premium of £5 is paid on entering, which is returned in the case of all nurses who finish their training. As a rule they go on with it."

"Do you get a sufficient number of applicants?"

"In August I had 32, but the number varies. We are always able to select the candidates who seem to be the most suitable, and I think that we are fortunate in getting a very promising selection of applicants."

### THE NURSES OF THE DAVID LEWIS NORTHERN HOSPITAL, LIVERPOOL—*Continued.*

### Promotions from Within.

"I conclude that you do not pay a salary at first?"

"Not until after a nurse has been here six months. The salary then starts at £12 a year, and is raised to £15 for the second, and third year £20. In the fourth we pay £26, and, if they stay on the private staff after that £30. We always choose the sisters from the private staff and not from outside. It is encouraging to them. Generally speaking, our sisters obtain good appointments subsequently. Two are just now applying for admission to the Army Service, and one to the Indian Service. Uniform is found, but the wearing of it out of doors is optional. In practice, however, a great many nurses wear it as a matter of convenience."

### Abolition of Daily Scrubbing.

"When are the nurses due in the wards?"

"They are called at 6.30, have breakfast at 7.15, and then the whole of the day staff, including, sisters, go on duty at 8. Probationers here are not required to do sweeping."

"Then you have more than the usual number of ward-maids?"

"Yes; since I have been here I have started resident wardmaids instead of daily scrubbers. Practically, they are housemaids. At all events the result is that the probationers have less menial work than formerly. In many hospitals want of adequate accommodation prevents the engagement of a number of wardmaids, but here we have plenty of room."

"When did you introduce the system of resident ward-maids."

"At the beginning of this year. I commenced with eight, and the experiment has been quite satisfactory all round. My point is that the arrangement gives the nurses more time for their nursing duties."

### Off Duty-Time.

"How long do they remain in the wards?"

"The probationers leave at 8 P.M., and the assistant nurses, who are in their third year at 9. The dinner hour for the probationers is 11.30, and for sisters 12.45. The probationers are off duty from 2 to 4; one day a week from 4 to 6; every other Sunday from 2 to 9.30, this gives them half a day off once a fortnight. The assistant nurses and sisters are off duty alternate evenings from 5 to 9.30; and if not required by the medical staff, they have a little time every afternoon. Every other Sunday they are off from 2 to 9.30. Every member of the nursing staff gets a full day once a month and a month's holiday in the year, the whole of the 28 days being taken at one period."

"When do the night staff come on duty?"

"At 9 P.M. Prayers are at 9.30 and the day nurses get half an hour in their sitting-room before bed-time. The night nurses leave the wards at 8.45 A.M., have dinner at 9 and are off duty until 12, except two days in the week, when they go off from 5 P.M. until 8 instead. With the night sister there are on duty one nurse in each ward and two in the children's ward. One is also kept in reserve for emergencies. These are in their second or third years."

"What is the number of beds occupied?"

"A hundred and eighty. There are 23 to 25 in the medical and surgical wards, and 25 in the children's wards. Two of the wards are circular. With more money we have room for 212 patients."

### The Night Nurses' Diet.

"Do you find any difficulty as to the diet of the night nurses?"

"It is not easy to give them as much variety as for the day staff, but we vary their ward meal as much as possible, alternating meat with fish and rissoles. The ward meal is at midnight, but they have breakfast when they start in the evening. As I mentioned, when we went through the Home, the night and private nurses sleep in the hospital at the top of the building; they have separate bedrooms."

"With regard to other features of the hospital, you have a nicely-appointed chapel, and a large room which I conclude that you use, among other things, for entertainments to patients in the winter?"

### The Lectures.

"Yes; and for lectures to the nurses. It may be appropriate at this stage to allude to the theoretical teaching. The honorary Medical Staff take great interest in the training of the probationers. Lectures, medical and surgical, are given in turn. They begin at the end of October, and continue until the examinations, of which there are three. I give a course of lectures on nursing, and the house surgeon gives a course of lectures on instruments; the home sister takes classes on the lectures, and sees that the nurses quite understand them; the medical sister gives lectures on testing; and the housekeeper-sister on bandaging. At the end of the fourth year the certificate is presented, so that the recipients are all members of the private staff."

### The Private Staff.

"You utilise the private nurses in the hospital?"

"Always, when they are not engaged. Of course, a private nurse has a reasonable time off duty when she has finished a case, but then, unless she is sent to another case, she comes into the ward. The private nurses take the place of the sisters in their holidays. It is a good preparation for them."

"Are their services in request outside?"

"The demand is considerable, and from various parts of the country. We have some engaged now in London and Torquay, possibly by people who have previously lived in this district.

### Theatre Work.

"How long does a nurse go on theatre duty for a stretch?"

"An assistant nurse for 12 months, and a probationer for three months. This is done in order that as many nurses as possible may gain a knowledge of theatre work. As to other departments, there is always a sister and assistant in charge in the kitchen at the top of the hospital, and we are rather proud of our laundry in the basement, which has all the latest appliances and is a great convenience."

"I see that there is a tennis court for the use of the nurses."

"Yes, that is the only kind of outdoor recreation provided for them. They can get so easily on the dock-wall, which is very pleasant, across the water to Cheshire, or spend half an hour on a steamer, that nothing else is required. Indoors there is the library, and there is also a piano."

### The Home.

"That suggests the Home, which seems to have been admirably planned."

"The idea was that it should be away from the hospital and yet be connected with it. There are several bedrooms, as well as box-rooms and bath-rooms, and the housemaids sleep in the Home.

"There is no difference, I think, in the furniture of the sisters' and probationers' bedrooms?"

"None whatever; each nurse has a bedroom to herself, with all necessary articles of furniture; the floor is polished, and the centre is covered by rugs. Every room has a fire-place, but is heated by the radiator and lighted by electricity."

"Are they allowed to make tea in their rooms?"

"All the meals are served in the nurses' dining-room in the basement of the hospital; the sisters, the assistant nurses, and the probationers have a sitting-room to themselves. The probationers' sitting-room contains both an organ and a piano. There is also a very good room for bicycles. During the time the rebuilding of the hospital was proceeding the nurses found it difficult to get out of the city, and the Committee purchased a couple of cycles for their use. These hospital bicycles can be hired by any nurse for half-a-crown a season.

### The Religious Question.

"Is there any religious difficulty?"

"None at all. We scarcely get any applications from Roman Catholics, but there are several Nonconformists among the probationers. They do not, however, object to attend the services in the chapel. The nurses are required by the rules to attend."

"I gather that the cases are of a very representative character."

"There are a great many surgical operation cases, and also a large number of serious accidents not involving operations. In the medical wards there are many cases of pneumonia and enteric fever. As I pointed out, there are open-air balconies, which the patients greatly appreciate.

### Varied Experience.

"Our method," continued the matron, "is to change the probationers about as much as possible in order to give them the benefit of varied experience. The assistant nurses have three months in turn respectively in the medical, surgical, and children's ward. I am glad to take nurses who have been in a children's hospital because they know the rules and understand the discipline. Certainly, preliminary training is far from telling against those who desire to join our nursing staff."

### Nursing at Kaiserswerth.

BY A SPECIAL CORRESPONDENT.

A great wish to visit Kaiserswerth has at last been fulfilled, and it may interest English nurses to know that it is by no means a formidable undertaking. A trip through Holland and into Germany as far as Düsseldorf, then *viâ* electric tram or rather three electric trams to Kaiserswerth. It is advisable to be prepared with a few German sentences for purposes of inquiry, as I found some difficulty in discovering which direction to take, so few officials seemed aware of the existence of Kaiserswerth, or it may have been that my pronunciation was at fault; however, after following contrary directions up and down a shady avenue in Düsseldorf, I at length discovered a man who was going to the same destination, and so my anxiety ended. On alighting from the tram the traveller sees an imposing red brick building, standing in spacious grounds; to the right is a smaller building, which is the Krankenhaus or Hospital. The former, bears the name of "Mutterhaus," and is the official headquarters of the Deaconess Institution, founded by Pastor Fliedner between the years 1830-1840. The original hospital was opened in October 1836, practically with neither funds, nurses, nor patients. The founder could neither secure sympathy nor assistance from the leading burghers of Düsseldorf, therefore he was compelled to depend on his own very slender means for the equipment of the much-needed hospital. The furniture at the opening consisted of a table, some chairs with rickety legs, a few worm-eaten bedsteads, and various damaged knives and forks. The first patient was a Roman Catholic servant-maid. As the hospital at Kaiserswerth was the first training school for nurses in Europe, it may be asked who was the first trained nurse, and where did she get her training? The founder, Pastor Fliedner, had obtained the services of a physician named Reichardt, whose daughter Gertrude had for some years assisted her father, and had been taught by him the essential principles of nursing. She became the first nurse, and in her turn taught others what she had learnt and so started the training school of which Florence Nightingale heard from Mrs. Elizabeth Fry, who had visited Kaiserswerth in 1840.

### Florence Nightingale's Training.

From this small beginning sprang an institution which has sent forth Christian nurses into all parts of the world. By the end of the first year there were 40 patients and seven nurses. Of course in those days nursing was more difficult than it is to-day. Without suitable accommodation either for nurses or patients, without necessary appliances for the treatment of the sick, without the scientific methods of the present-day order, it was yet possible for the obscure little hospital at Kaiserswerth to give Florence Nightingale such a training as fitted her for the great work to which she was afterwards called. It was not without some difficulty that she secured admission as a probationer, as the work was hard, and Pastor Fliedner feared that she would not be strong enough. Eventually, however, he relented, and "the foreign lady," who was so eager to learn, won the esteem and respect of all her fellow-workers, and the physician of that day learnt to value her skilful touch and kindly heart and took especial pains to teach her what she desired to learn.

### Kaiserswerth of To-day.

The modern hospital at Kaiserswerth receives about a hundred patients, each general ward holding from 10 to 12 beds; the bedsteads have wooden sides, and are by no means as easily taken to pieces as are ours in England; the quilts and pillow-cases are made of a check material, red and white. At the head of each bed is a black slate, whereon is inscribed the name of the patient, date of admission, and nature of diet; also a little satchel which holds the temperature chart. The treatment card is not left within reach of the patient; surely a wise precaution. The lockers are of painted wood similar to our own, and a chair stands at the bottom of each bed. Each ward is warmed by a closed stove, and otherwise is well lighted and ventilated. The walls are painted a pale green and the floors are polished. The male patients wear cotton striped suits (when out of bed), provided by the hospital.

### The Patients and Diet.

There are three classes of patients, first-class who pay 6s. a day; second-class, 3s.; and the third-class about 1s. per day. If a patient is unable to pay anything he receives treatment gratis. The meals are served as follows—at 6.30 coffee and milk and bread; at 9.30, soup or coffee and milk; at 12, the midday meal according to the class of diet ordered by the

### NURSING AT KAISERSWERTH—*Continued.*

doctor; at 3 P.M., coffee and bread again; at 5 P.M., supper, which consists of soup and meat and vegetables for ordinary diet; and at 7 P.M. another light meal. From a glance at the kitchen and larder, the food looked very inviting; good soup, stewed fruits, and fruit jellies were awaiting consumption, and the plump, kindly Sister in charge of the kitchen appeared to be an adept in the art of invalid cookery. The cooking is mostly done by steam in immense boilers, but the meats are cooked in ovens heated by coke and coal. Tiled floors and partially tiled walls give a cool, clean appearance to the kitchens, of which there are two, a separate one for the preparation of coffee and the heating of milk. This latter entails considerable labour.

### Regulations and Training.

The nurses come on duty at 6 in the morning, at 7 they have coffee and bread and butter, lunch or second breakfast at 9, and dinner at 1 P.M. Coffee is served at 3.30, and

Uniform of a Kaiserswerth Nurse.

supper at 7 P.M. Nurses go off duty about 9 P.M. They have no regular recreation hours, but, when they can be spared, half an hour in the fresh air is permitted. The afternoons are devoted to study or attendance at lectures given by the pastors, medical officers, and matron. Holidays, as a rule, come once in two years, but more often if necessary. The probationer, or probe-schwester, must be between 18 and 40. She serves her probation until she is considered qualified to become a Sister. The period varies according to the quickness and intelligence of the would-be Sister. She must be a Protestant, and enter the hospital with the intention of becoming a deaconess. Usually a probationer spends the first year at Kaiserswerth, and afterwards is sent to some larger hospital to gain further experience. Frequently the course of training lasts from five to six years, and may include lessons in cookery, housekeeping, laundry work, sewing, etc. The nurse, when qualified, may become a district nurse, work in a hospital, or be sent to private cases. She is never sent to an infectious case without her free consent, but so far a refusal has never been known, nor is she sent to foreign countries against her will, but within the borders of Germany she must consent to any arrangement made by the authorities of the Institution. No salary is given, but the sum of five pounds a year is granted for pocket-money.

### Indoor and Outdoor Uniform.

The uniform consists of a dark blue dress with tiny spots; it is of washing material, with a very full skirt and yoked bodice, a blue cotton apron, round white collar, and spotted muslin cap with quilled border and narrow strings. The shape of the cap is stereotyped, and permits of no individual touches. Every nurse's hair must be parted in the middle and neatly brushed; should she by nature possess curly locks, then pomade is forthcoming to reduce them to the desired condition. Such frivolities as Hinde's hair-curlers are unknown among the deaconesses. The outdoor dress is extremely quaint, an early Victorian style of drawn silk bonnet with short black silk veil, this is worn over the muslin cap, a uniform cloak with short cape, or black cashmere shawl, worn with the point in the middle of the back; and for Sunday wear a dark blue merino dress with a small cape of the same material, the cape being a trifle longer than those worn by our army nurses. The Sunday dress is renewed every five years. Two cotton dresses are supplied yearly and about four caps. The probationers wear a different shaped cap, rather more severe in style, and black dresses for Sunday, which they provide themselves; a short black veil is likewise worn over the cap for out-door walking. It is curious to see young fresh faces under these severe caps. Apparently the Sisters and probationers are perfectly happy and desire no change in their dress, though from the beginning of the Institution, more than 60 years ago, no modification has been made.

### The Nurses' Quarters.

The nurses' dining-room is a large well-appointed room, and beautiful pictures adorn the walls; the latter being decorated with an ornamental dado. Opening out of this handsome apartment is an equally commodious sitting-room, furnished with comfortable chairs, writing tables, etc. A piano and a harmonium have also been provided for the recreation of the Sisters and nurse-probationers. The unusual elegance of these rooms is due to the benevolence of friends of the Institution, and not to reckless extravagance on the part of the Institution as was most carefully explained by one of the Sisters. A large balcony overlooking a garden seemed to me an ideal place for quiet reading and rest, but Kaiserswerth nurses do not have much time for either. The large dormitories provided for the nurse-probationers are divided into cubicles, cool linen curtains separating one from the other, but though no space is lost, everything essential to comfort is packed into the cubicle which resembled a ship's cabin for neatness and order. The hospital is lighted throughout with electric light.

### A Home of Rest.

Not far from the main building is a home of rest for infirm and aged Sisters and nurses. Here they retire from active work and spend a peaceful old age amid the surroundings to which they have so long been accustomed. Each

Sister has a separate room provided with every comfort and has no further anxiety with reference to the future. She will be cared for to the end of her days and receive the loving attention to which a long life of ceaseless self-denial has certainly entitled her. One old Sister of 93 still goes occasionally to church, and takes the most lively interest in all that passes around her. The uniform once donned is never put off. This is of great advantage in many ways as it simplifies the question of holidays considerably, lessens the drain upon the nurse's private expenses, and insures to the wearer civility and attention wherever she goes. Moreover it is never copied by any private individual or other Institution. More than 1,000 dresses are made every year, and one Sister spends practically all her time in the making of Kaiserswerth bonnets. No nurse is allowed to accept gifts direct from patients, she must hand them over to the matron who returns them to her as Christmas gifts. Constant demands are being made for trained Kaiserswerth nurses; they are now at the head of hospitals in many eastern countries, such as Jerusalem, Cairo, Beyrout, South Africa, and India. At Alexandria they have entered the harems to nurse the great Mohammedan ladies who are never permitted to see the face of a male doctor.

### The Operation-room.

But I have not yet fully described all the arrangements at the Kaiserswerth hospital. Being a surgical nurse, I was greatly interested in the operation-room and its various details. These were explained by the operating surgeon himself. Patients are anæsthetised in an adjoining room by a fully-qualified Sister, and then conveyed to the operating-table, which is very lightly constructed, movable at both ends, and very easily disinfected. An immense plate-glass window to the right of the table throws a splendid light upon the surgeon's movements, while small glass ambulance tables stand near at hand, with glass trays for instruments, glass kidney dishes, and a light stand holding various antiseptic solutions, all plainly labelled. Upon one of the walls just over the doctor's lavatory basins are directions for disinfection of the hands; this is writ so large, that it is impossible to omit the ceremony. Five minutes at the very least must be spent in brushing and general purification of the hands. I inquired if these instructions were perused daily, but a young assistant surgeon assured me that they all knew them by heart. A glass instrument cupboard is let in the wall between the two rooms, and can be opened on either side, a most convenient arrangement. The floor is of stone, and undergoes frequent disinfection. I was shown a powerful electric battery, also an arrangement for the application of local heat in rheumatic cases. Antiseptic treatment is evidently very thoroughly carried out at Kaiserswerth, and each new probationer is taught its importance.

### The Sister Dispenser.

The dispensary is in the hands of a qualified Sister who has passed a State examination and does the dispensing for all the branches of the institution at Kaiserswerth. I was greatly struck with the extreme orderliness and cleanliness of this department. The poison cupboards were all doubly locked, a precaution insisted upon by Government inspectors who may pay a surprise visit at any moment. Every bottle seemed to shine forth the praise of the Sister dispenser who could do so much work and yet leave so little trace of it. Even the little scales in which deadly poisons were weighed were duly labelled to avoid all possibility of accident.

### The Children's Wards.

The children's wards are in a separate block and rejoice in a charming covered balcony where the little patients all day long enjoy the fresh air. In this building, at the time of my visit, were 30 children, but there are beds for 60. Many of them were suffering from rickets, which in Germany is called the "English disease." It was sad to see so many puny, white little faces in the quaint little wooden bedsteads, but every effort was made to amuse them by their nurses. Rocking-horses were in full swing for the convalescents, swing-boats, doll's-houses, picture-books, in short all that could be desired to gladden the eyes of little people. Some little boys were attired in cool-looking cotton suits, and suffering only from eczema in the head, were able to enjoy life to a great extent.

### The Ward Kitchen.

In the ward kitchen, which was presided over by a flaxen-haired probationer in her quaintly stiff cap, were shining crockery, knives and forks, etc., and receptacles for the same of amazing orderliness. A delightful dining-room for the convalescents, with low tables and benches for the smaller children, evinced the thoughtful care which had arranged every detail for the comfort of the tiniest child, nothing seeming to have been forgotten. Another building was set apart for sick Sisters so that they might have necessary quiet and attention without the inevitable associations of an ordinary hospital. Each patient has a separate room, comfortably and brightly furnished with all that weary, suffering woman could desire to look upon. Yet another house is set apart for patients suffering from nervous diseases and brain disorders. Quite a small park surrounds this building which has accommodation for 150 patients. These also are divided into three classes and pay respectively £75, £60, and £45 a year. Incurable cases are received and are treated with unfailing care and kindness by a band of deaconess-nurses.

### Funeral of a Sister.

During my stay at Kaiserswerth, the funeral of a Sister took place, and I shall never forget the impressiveness of the ceremony. The flower-covered coffin rested temporarily in the beautiful church, through whose stained-glass windows streamed the bright sunshine upon the throng of black-bonnetted Sisters, whose heads were reverently bowed as they listened to the solemn words of the Burial Service read over one of their own number, by their own beloved pastor. Quietly and sadly the mourning sisterhood followed their comrade to her last resting-place in the quiet God's acre, where the soft summer breezes sang a gentle requiem over the newly made grave. In this sacred spot lie most of the Sisters who have spent their lives in the service of the Institution, and it is hallowed to the living community still further by the graves of the revered founder, Pastor Fliedner, and that of the first superintendent or head sister. A more peaceful burial-place one cannot imagine.

### The Queen of Nurses.

It was very pleasant to learn that the letters of Florence Nightingale are preserved with loving care, and to know that her memory is still fragrant, though many of her fellow-workers have gone to the Silent Land. I was shown the identical rooms which she occupied by the white-haired Sister who now uses them, and noticed that she considered it a privilege to do so; some American nurses visiting Kaiserswerth were equally interested in seeing the surroundings in which the "Queen of Nurses" received her training, the results of which have been so far-reaching in their influence upon the lives and character of thousands of women in many lands beside our own beloved country.

### Lepers of the Transvaal.

BY AN ENGLISH NURSE.

On the lower slope of a hill, about seven miles to the west of Pretoria, lie a number of buildings forming quite a large village, surrounded on three sides by high hills, with a wide open plain in the front, affording a fine view of the city. Strangers entering this village for the first time, seeing its tidy, well-kept roads, its pretty little houses and gardens, and noting its general air of prosperity, would not guess that this is the abode of the most unfortunate of all human beings, for every cottage is inhabited by lepers.

THE LEPERS' GARDENS.

Here are old men and women, youths and young girls, children of all ages and all shades of colour, every one afflicted with this hideous disease. They will greet visitors with a smile, the more sensitive perhaps trying to hide their deformities, but most of them are so proud of, and eager to show, their gardens that they forget to be shy. The state of these gardens is the more marvellous because they are often made and attended to by those who have very little left in the way of either hands or feet. One blind Basuto youth has outlined his piece of ground with empty milk tins; they are placed one after the other with wonderful accuracy, forming quite a pretty edge, to keep the soil in its place. It is marvellous what these people are capable of doing. The whole work of keeping the gardens tidy; planting and gathering in of mealies, barley, and other crops; cutting and stacking hay and coarse grass, etc., for the cattle; herding the latter; keeping the roads in repair; and all the thousand and one small things which require doing in a big place like the Leper Asylum, are done by the patients themselves. None of them is made to work, and only those who are physically fit are allowed to do so. They are all paid for the work they do—a small sum, of course, as the Government provides every one of them with necessaries—but their small earnings help to procure them small luxuries and in some cases to buy food for the families of those who are married, this being especially the case with the white men. Each patient must keep his or her house clean and tidy; if any of them, white or coloured, are unable to do so, another patient does it, and is paid for the work. The rooms of the patients are very pretty, many of them taking much pleasure in keeping them so, especially the white women.

General View of White Male Quarters, with Houses of Officials, Leper Asylum, Transvaal.

QUARRELS BETWEEN TRIBES.

There are many natives, almost every tribe being represented. They are very clannish, each tribe cooking and eating their food together, and making friends with each other. Occasionally there are quarrels between members of different tribes, generally over the most trivial matters. For instance, a Basuto native was making mealie porridge one day in the compound, a Mashangaan happened to pass the fire and pot, "Ah!" he said, throwing a sly glance at the pot, "there's not salt enough in that mealie pop." The Basuto snatched the spoon with which he was stirring the porridge out of the pot, and attempted to strike the Mashangaan, but not having much left in the way of feet, he overbalanced and fell, burning his face with the hot porridge-spoon, whilst the other boy ran away laughing. Presently he returned, and looking at the pot, said again that there was not sufficient salt there. The Basuto was ready for him this time, he had a piece of iron in his hand, with which he hit the Mashangaan a fearful blow on his cheek, laying it open. This was enough, the Mashangaan closed with him, and there was a big fight, natives of each tribe coming to the help of their "brothers," yelling and making a huge hubbub. Finally the matron succeeded in making them quiet, inducing the two boys to say they would not do it again. This happened over three years ago, yet to this day those two are not friends. (N.B.—A male native is a boy, no matter what his age may be.)

### The Leper Children.

There are many children, dear, chubby little things. They play and amuse themselves much the same as other children do, and often run after the matron, begging for sweets, etc. Alas! some of them have already lost fingers and toes, their faces, too, being badly marked in some instances; one little white girl, who is only nine years of age, would move the most hard-hearted person to pity her. She has the face of quite an elderly woman, and is constantly suffering with sores. Yet she is rarely heard to murmur, and is such a sweet gentle child that she has won the affection of everyone round her. In such a place as this there must be some rules and regulations, but they are very few indeed, every effort is made to make the lives of the inmates as much like being in their own homes as possible. Their separate rooms help towards this end considerably, giving them greater privacy than they could possibly have were they placed in wards, a fact they very much appreciate. In the white female quarters the houses are built in blocks, containing four rooms, opening on to a stoep, *i.e.* verandah, with a smaller room behind each of the larger ones, making a living-room and a bedroom for each patient. The white male quarters are built in a similar way. In the white married quarters each house contains two front rooms, each opening on to the verandah, and a kitchen at the back with a door and steps. In the native compounds, both male and female, each block of four rooms contains eight inhabitants, two to each room, each block having a kitchen behind, which is used by all. There are good bath-rooms, with hot and cold water, many of the patients taking medicated baths as part of their treatment.

White Married and White Single Male Quarters, Leper Asylum, Transvaal.

### Recreation for the Patients.

There are recreation-rooms for each portion of the Asylum. As many entertainments as possible are given to them, which they very much appreciate. Very specially do they like going to a picnic. A spot has been chosen for this purpose about two miles away, where some trees are growing, really around an old disused Basuto kraal. They are taken there in ox-waggons and ambulances, accompanied by a cart with provisions and a water-cart, starting very early in the morning, and not returning until sundown, very tired, but happy in the thought that they

LEPERS OF THE TRANSVAAL—*Continued.*

have had heaps of nice food, and perfect freedom for one whole day, in a spot where they cannot see a vestige of the Asylum. A fund has been started in Pretoria by means of which these picnics and entertainments are provided. It does not, however, allow of very frequent pleasures, there are so many patients to provide for; but if those who give the means to make these pleasures possible to sorely afflicted people could only see how the monotonous lives are brightened thereby they would feel amply repaid. All the patients, both white and native, are extremely fond of music and singing. Many are the requests to the matron for concertinas, zithers, mouth-organs, violins, banjos, etc., only a few of which she is able to supply. There is a very nice organ in one of the recreation-rooms, which is a source of great and continual pleasure to the lepers. Some of them sing very well; it is quite pleasant to listen to the volume of sound which goes up at the services. There is a missionary for the natives, who comes to them nearly every week from Pretoria. They enjoy his visits a good deal, and hold services of their own when he is not there. The church for the white people is a very curious building. It is as though a big slice had been cut out of the middle, so that the place where the patients sit is entirely separate from the part occupied by the preacher. Many comical incidents occur daily; many odd speeches are made by the patients, who are entirely innocent of any intention of being funny. Natives always note anything which is peculiar in the gait or appearance of those with whom they are brought into contact, and amongst themselves give white persons very characteristic names, every official at the Asylum being aptly described by them in this way. It sounds odd to hear quite grown-up men and women, if they wish any special favour, saying to the matron that she is "the only mother they have now," and is, moreover, their "old missis," these being excellent reasons in their eyes why the favour should be granted.

Visiting Days.

The patients are allowed to have visitors, so on Sundays there is usually quite a number of relatives and friends seated in small groups on the ground near the church. They arrive often by six in the morning, and bring their kettles, cooking-pots, cups, plates, etc., and their food with them, making fires in the true picnic style, and remaining all day. They arrive in all sorts of conveyances—donkey-carts, mule-carts, and occasionally in ox-waggons. They do not touch their leper relatives; they only sit on the ground a little distance away and talk to them. It is a sadly pathetic sight. To most people it would be dreadful to see their nearest and dearest without being able even to touch them, yet it seems to give these sufferers pleasure. It is something to be able to talk over with their fellow patients during the ensuing week all the news of the outer world which their friends carried to them on Sunday. This Asylum for Lepers, which is one of the oldest institutions of the Transvaal, was originally at Daspoort, much nearer to Pretoria than the present one. The old one, which was found to be quite inadequate and very dirty, was burnt by order of the Boer Government as soon as the present Asylum was in a fit condition to be inhabited. It is well planned and well built, so the Boer Government did one good thing with the Uitlanders' money when they had this place made.

Hopes of Recovery.

All the patients hope constantly for recovery, even those who are terribly disfigured and deformed, most of them being very eager for treatment. Very few persons, except the patients' own friends, are aware of the existence of this Leper Asylum; still fewer know the amount of leprosy there is in the Transvaal. Yet this Institution, with its 300 inhabitants, each member with his own desires and hopes, each with his own burden of suffering and sorrow, is a most interesting place to see, and should not be missed by the many visitors who now find their way to Pretoria.

### Obstetrics in a Caravan.

BY A DISTRICT NURSE.

The picturesque wide street of the village of P—— is annually invaded by a time-honoured fair, much looked forward to by the younger part of the populace, but, fortunately for the older residents, only lasting one day.

About 5 A.M. the day following the fair, I heard a loud knock, and going down, saw a fine-looking young gipsy-woman, who begged me to come at once to her friend in one of the caravans, which had already, it appeared, started off, but had had to be brought back again within reach of assistance.

It was quite a novel experience to me to find myself mounting the steps of a caravan. Arrived within the tiny space I found my patient, though the mother of about eight children, had evidently sent for me too soon. So I had plenty of time to look round, and also to accept the offer of a cup of tea—a great boon—as, in spite of open-door and skylight, the atmosphere was decidedly warm, and besides myself and the patient, two friends insisted on remaining the greater part of the time—five hours!

Everything was very neat and clean. From the polished brass rail above the tiny cooking-range hung a suit of baby-clothes airing, while the mother had saved a garment of real linen for her own wear.

On the left side of the door on entering was fitted a cupboard containing cups and saucers, bread, tea, etc., then came the range, in which a good fire was burning, though a warm morning; then a slab which served as table, under which, concealed by a cretonne curtain, was a space where various household articles were kept. Across the end of the van opposite the door was the bed—like a berth on board ship—so high from the floor that the occupant had to mount by means of the only chair, but fairly roomy. After I had been some time in the van, I found a child in a so-called bed underneath the principal one, and quite concealed by a valance-curtain. The other persons attached to this abode were outside with the father; when I asked if they did not find their sleeping quarters rather suffocating the mother said, "Oh no, they were used to it." All down the other side of the van ran a locker, which served the double purpose of a wardrobe and a seat, while the walls and ceiling were also utilised for hanging purposes. There was even an attempt at ornament, one or two pictures, and on the mantel-shelf, kept in place by a rail, were some China knick-knacks. I was told they had a much better van "at home" where the rest of the family were.

Though an interesting experience, I was not sorry when I had seen the mother comfortably ensconced in her lofty bed, with a fine baby-boy, to leave my warm quarters and go home to breakfast. There seemed to be no lack of money, and a contribution to the fund was cheerfully given. After a day or two the van proceeded by easy stages on its way.

I was amused at being almost invariably addressed as "my lady!"

### The "Stage Army" Dictionary.

It is rumoured that the promoters of the "Stage Army" have a new Dictionary in preparation. We gather from their paper that the following are a few definitions which this Dictionary may contain :—

*A fact is* "a scurrilous misrepresentation."

*Exposure applied to a group of adventurers is* "a vicious attack."

*Truth is* "coarse abuse."

*A representative body is* "class legislation."

*Refusal to be ruled by adventurers is* "unprofessional policy."

*The republication of palpable falsehoods which have often been exposed is* "the expression of the opinion of the Leaders of Nursing" (self-constituted).

The publication of a few wholesome truths in the article entitled "Class Legislation" which appeared on page 5 of the *Nursing Section* of The Hospital for October 1st last has put the Generalissimo of the Stage Army into a high fever, which is said to be accompanied by head symptoms that threaten to destroy the Generalissimo's reason. This is very sad; but facts have before proved destructive of adventurers, independent of sex.

### The Duchess of Albany at the National Hospital for the Paralysed.

BY ONE OF THE NURSES.

At breakfast tongues wagged fast and furious, the topic being the coming of "Our Duchess." Beds were made, wards swept, lockers dusted and polished, and the "brasses" became "shiny" beyond compare.

At eight o'clock Sister came on duty, and the decorations were proceeded with. The morning passed quickly, and by two o'clock everything was in order. At this time the appearance of a counterpane that would go awry, despite all efforts, was sufficient to make staff nurse and probationer pathetic.

Three o'clock came and the majority of the nursing staff were gathered together in the hall. Exactly facing the door the sisters stood, in their dark-blue uniforms. On the staircase above them, the staff nurses and probationers were congregated in all their glory of spotless cotton frocks—the former in light blue, the latter in pink. The visitors arrived quickly, and took up their respective positions. At half-past three a commotion outside heralded the arrival of the Duchess, and Princess Alexander of Teck.

The Chairman went forward immediately, and greeted them. His little daughter, curtseying prettily, presented a beautiful bouquet of pale pink flowers, with lilies of the valley, and maidenhair fern to the Duchess, who smiled kindly and shook hands with the little maiden. Next the chairman's little boy stepped forth, and presented a charming white bouquet to the Princess. Then the members of the Visiting Staff were presented to the Duchess. Shaking hands with the matron, Miss Vernet, she caught sight of the smiling faces on the stairs. With a smile she bowed.

"What a sweet smile," whispered an enthusiastic junior.

"Hush," said the stern voice of a senior.

"The Duchess is wearing a royal purple velvet gown," still whispered the irrepressible junior. Then silence reigned. That junior wonders if there is an affinity 'twixt smiles and velvet gowns.

Down a wide corridor, across the grounds and into the operating theatre. After a short prayer by Bishop Welldon, the Duchess, in a few well-chosen words, declared the operating theatre open; after which the visitors thoroughly inspected the theatre.

In the wards here flowers bloomed, and palms raised their stately heads. All the wards were charmingly decorated. One was a mass of white and yellow chrysanthemums, another pink and white. A third was gorgeous in scarlet flowers and green foliage. A fourth looked beautiful, with mmense palms, huge yellow chrysanthemums, and variegated foliage standing in tall quaint brown pots.

Yet another, the children's ward, was entered through a bower of Michaelmas daisies, which peeped shyly out from their nest of green. There were flowers here, there, and everywhere. White chrysanthemums, marguerites, and pink roses galore. The coveted cots were "The Duchess of Albany." They looked very pretty with their white canopies and pink flowers. In one of them reposed Victoria —— (called Vic.) æt. 3½ years, queen of the ward. She had captured our hearts long, long ago, and we were her most loyal subjects. Vic. as a rule behaved well. She had been told that the "Duchess" was coming and had been clearly puzzled thereby.

She knew by subtle instinct the difference between nurse and sister. She had been spoken to most graciously on more than one occasion by a lady whom both sister and nurse addressed as "Matron." But a Duchess!

We had tried to teach her to say "Good afternoon, Duchess," and to make a little "bob." But on the arrival of her Royal Highness Vic. settled the delicate question of etiquette in her own way. Hardly had the visitors entered when there was a patter of little feet and Vic., barefooted, clad in a pink "nighty," stood before them. In her hands she held a little bunch of violets. She lifted them up to the Duchess saying simply in her childish treble, "Please lady." The "lady" stooped with a smile and said, "What a sweet wee thing!" Vic. walked calmly to her cot and gazed around as if the presentation of a floral tribute to a duchess were an every-day occurrence.

In another ward a little girl on being asked what ailed her said, "Please ma'am I'm 'paradised'" (she meant paralysed). The Duchess and Princess visited several wards and won all hearts by their sympathy and gracious words.

At 4 45 p.m. we were all seated in the chapel. At five o'clock the Duchess and the Princess entered; shortly after the service commenced. The choir, with two exceptions composed entirely of nurses, sang appropriate hymns and an anthem "Ye shall Dwell in the Land" (Stainer). Bishop Welldon gave a short address, touching on the great debt we owe to those who spend their time in scientific research for the alleviation of human sickness.

The soft light fell upon the crimson blossoms which were strewn upon the altar, and upon the "first fruits" of the season; ascending, it dwelt lovingly upon the symbol of our faith, touching it to living gold. Still further it crept, until the windows were bathed in its soft radiance, merging the wonderful reds, greens, blues, and purples into indescribable beauty. As the solemn words of the Benediction were pronounced the light reached the central window, lingered there; and, lo! it was the Christus, with hands lifted in benediction, seeming to echo the words of so long ago, "My peace I give unto you."

And so the light remained.

After an interval of silence "God save the King" was played on the organ, and the visitors walked slowly out.

Once more we had a glimpse of our Duchess as she drove smilingly away.

And we went back to our wards proud that we had that day met a gracious lady who despised not the offering of a little child.

### Nurses for the Middle Classes.

### A CHAT WITH MISS THORNE.

Having seen the statement in the current number of the *British Medical Journal* that a philanthropist had volunteered to provide the necessary capital for establishing visiting nurses for the middle classes, I called on a lady who has been engaged in this kind of work in Bloomsbury for several years—Miss Thorne—and asked her for an expression of opinion on the matter.

"I should like to write to that philanthropist," was her remark, as I showed her the paragraph in question. "He seems to think that the idea is a new one. I thought so myself when I began, but I found that there were several others in the field."

"Do you think that there is a need for it?"

"I think it is a form of nursing that should be extended. After four years' experience my partner and I find that people are very glad to know that they can have a nurse who does not want to be put up in the house. That is a great boon to many people, especially those living in boarding-houses and hotels."

"Why did you take up this branch of work?"

"I was a Queen's Nurse at the Metropolitan Association Nursing Home, Bloomsbury, and at that time I thought that I had a mission to the poor, and that only the poor ought to be nursed. But as I grew older I came to see that anyone who was ill needed nursing, whatever their rank in life, and that there was often just as much need of it among what are called the middle classes as among the poor themselves. Now I think I am fond of all human beings; the fact that they are ill is enough for me. All the same, I love the poor. Some of the happiest years of my life were spent in Darlington among very poor people; that was ten years ago. I am also a midwife, and I have had several midwifery cases in this neighbourhood. That is a department of work that could be greatly increased, but of course a nurse who undertakes it must be prepared to be called up at night to attend the confinement, and to visit the case twice a day afterwards."

"In ordinary cases do you confine yourself to visits?"

"Yes; it is necessary to draw the line there. Sometimes a case may be a daily one to begin with, but, becoming more serious, requires constant attention. Then we retire in favour of the private nurse. I do not think it would do to overlap."

"What kind of patients do you mostly attend? The 'poorer clergy, tradesmen, and clerks,' to quote the paragraph?"

"Very few clergy, but a fair number of tradespeople and their assistants and clerks, including many young men in lodgings. They are not hospital cases, neither can they afford to let their weekly expenses run up to five guineas, as they would do if a private nurse were engaged. We do for them what the Queen's Nurses do for the poor, and they are thankful for it."

"Is there any risk of bad debts?"

"I think I have only had two bad debts all the time I have been engaged in this work; by asking for weekly payments, that risk is almost entirely avoided. Our fee of a guinea a week covers two daily visits, and we charge 12s. 6d. a week for one. We do not let the fees run on till the case is finished. We do not find a guinea a week too much, most people are willing to pay it."

"Do you restrict the length of the visit?"

"No, but it averages about an hour, unless it is an operation or anything special. I think the plan of charging so much an hour, say 3s. 6d., a bad one. It is not pleasant either for the patient or for the nurse to have to watch the clock and feel that the time is not being rigidly adhered to."

"Are there any special difficulties in this kind of nursing?"

"Not any serious ones. It would be better if one could begin work earlier in the day—say, starting out at 8.30, as the Queen's nurses do—but these people are not ready so early as the poor; and, moreover, they do not get hot water, etc., ready in the same way. One has to use a good deal of tact, for example, in a boarding-house, where there may be many stairs to run up and down, as to how much can be expected of the servants, or as to the advisability of doing some of the humbler duties oneself. Tact, indeed, is a prime necessity; indiscretion in talking about the patient to the people in the house might lead to much mischief-making."

"How many visits can you pay in one round?"

"I should say about five, considering the distances—we take in Holborn as well as Bloomsbury—and the possible waiting to see the doctor, if he wishes to meet the nurse; but if all the patients would not want to be washed at nine o'clock, one could arrange one's morning better."

"That," I suggested, "is where such a scheme as this would come in; there would be a larger staff to draw upon."

"In Bloomsbury," Miss Thorne replied, "there is enough work for two, but not for three, at present at any rate. I do not want to be selfish, and prevent others from coming in, but we have never yet refused a case except when one of us was ill. I think it is necessary to have two, as if the doctor sends in a hurry, and one is engaged, the other should be available. I always know where to find my partner, and *vice-versâ*. And I think that they should live together. But as to centres, and committees, and red tape. . . ." A shake of the head said more than words. "I think," Miss Thorne added, "that there must be great openings for this kind of work in rapidly-growing suburbs, such as Harringay, for example."

Summing up the experience of the past four years, Miss Thorne said that the life was a wonderfully free one, that a fair living could be made—£80 to £100 a year—and that, although warned at the outset that people would be difficult and disagreeable, her experience had been just the reverse.

### Irish Nurses' Association.

The Winter Session of the Irish Nurses' Association was opened at the Association Rooms, 86 Lower Leeson Street, Dublin, last week, by a social gathering of the members, of whom upwards of 125 were able to be present.

The evening began by a short business meeting, Miss Huxley, president, in the chair; the subject of discussion being affiliation with the International Council of Nurses. The members then adjourned for tea, after which a bright and attractive programme, arranged by Miss Haughton, lady superintendent of Sir Patrick Dun's Hospital, of tableaux, music, etc., was much enjoyed by all present. It has been arranged to hold two meetings each month throughout the winter for lectures, discussions, etc., and various doctors and friends have kindly consented to address the members on these occasions.

### Resignations.

St. Thomas's Hospital.—We are informed that Mrs. Swan, who has been for many years at St. Thomas's Hospital, has relinquished her position as assistant matron and retired from work. Prior to her departure a presentation of jewellery was made by the nursing staff and servants, by whom she was much esteemed.

### Everybody's Opinion.

[Correspondence on all subjects is invited, but we cannot in any way be responsible for the opinions expressed by our correspondents. No communication can be entertained if the name and address of the correspondent are not given as a guarantee of good faith, but not necessarily for publication. All correspondents should write on one side of the paper only.]

### CLASS LEGISLATION.

Miss Lavinia L. Dock writes from Berlin: An article from your paper in which my name appears having come under my notice, I feel constrained to ask your editorial courtesy for a few lines of your valuable space—a favour which I should hardly ask, and for the discussion of a subject in which I should hesitate to interfere, except in the interests of strict accuracy. The extract from an article of mine which you quote is assumed to have such an extraordinary intention, or inference, that I beg to state it was written for a public holding such a different standpoint from yours, as to registration, that its conclusions would be diametrically opposite from yours after reading the article in question. I may make myself clearer if I tabulate the two points of view thus:—

| *Our Standpoint.* | *Your Standpoint.* |
| --- | --- |
| State regulation of nursing education by the definition of a general basis, by examination, and by registration, is an educational reform, urgently demanded for the present, and a duty to posterity. | State regulation is "class legislation" . . . "will grant privileges to one-quarter of them and the other three-quarters are to be left in outer darkness." |

It is hard for the average American nurse who stays at home to realise the conservatism, privilege, monopoly, and short-sightedness which are to be found in high places in European training-schools, and my article was meant to show the difficulties against which English and German nursing reformers have to struggle. A commercial opposition is a less subtle antagonist. I am gladly correcting my inaccurate statement *re* "all" the London matrons. However, so far as "nine" are concerned, and this was the figure I gave, I think I was literally correct. But may I say that I could never be so stupid as to find it "abundantly evident" that nurses did not want registration. I consider nurses and matrons in Europe by no means always synonymous. To me the truly vicious class distinction seems to be that which complacently creates and maintains two classes or grades of nurse—one, an inferior grade, for the "poorer classes," and a superior grade, for whoever may get her. And, without State supervision of education, this is what happens. It is to make this hateful class distinction less possible for the future, by securing a broad, general, fundamental training for all nurses, that the power of the State is invoked by registrationists.

Lady Helen Munro-Ferguson writes from Novar, Ross-shire:—In the article which appeared in your issue of October 1st it is contended that those who advocate the registration of nurses do so in the interests of the rich, and that the trend and intention of the movement is to secure class legislation. Opposition to registration is represented *per contra* as a meritorious defence of the rights of the poor. The authority quoted in support of this view is a writer in the *Daily News*, who states that the present proposals for registration will "set up a standard which at one fell swoop will cast the nurses of the poor into the outer darkness of inefficiency." This is to assume that the nurses of the poor are, and must remain, a definite class of partially-trained women specially set apart for the lower classes; it is also tantamount to saying that merely to distinguish between the trained and untrained is to create inefficiency in the latter. So long, apparently, as the trained and untrained are covered by one name they are equally efficient, but, make it evident that they have not the same experience, and this equality vanishes. The case in point is that of the so-called cottage nurses employed by some county associations; these nurses generally receive a six months' training, and combine household work with the care of the sick. But committees who employ these nurses are fully aware of the limitations of their training; registration will enlighten them no further on this subject, nor will it alter the character of the services rendered by cottage nurses. If these nurses are doing an essentially useful work, the fact that they are not called "registered nurses" will in no way impair their usefulness or prevent their employment. All this, however, has nothing to do with the aims of registrationists; these are to raise the average standard of nursing throughout the profession. Registrationists contend that half-trained nurses are not good enough for anyone; that the poorer class need as careful and skilful treatment in acute illness as do the rich. They believe that the poorer the house the greater the need for educated and highly-skilled nurses, and they look to the great order of Queen's nurses (a registered body of fully-trained women), as the best and highest type of nurses for district work. Registrationists do not believe in one kind of nurse for the poor and another for the rich. They think that if any distinction is to be drawn it should be between acute or severe cases needing the highest professional attention, and those mild or chronic ailments which may safely be entrusted to less experienced hands. Registration, by making it easy for the general public to distinguish between trained and untrained nurses, would lead to such an adjustment of work; it would encourage local committees to secure the best nursing for bad cases, while anything which tended to raise the standard of training in hospitals and infirmaries, where the poor are generally cared for, would be as beneficial to them as to other classes. The writer further suggests that State registration would lead to class legislation for nurses, because it would be injurious to nurses trained in provincial hospitals and infirmaries, but it is difficult to see how this can be argued, for nurses will be registered on the evidence of their proficiency and knowledge, irrespective of where that knowledge and proficiency are acquired. At present, in the absence of all other safeguards, the public attach undue value to the certificate of London training schools, while infirmary-trained nurses are at a decided disadvantage in competing for employment with hospital-trained nurses. Registration will equalise their positions, and good training will receive its due reward wherever acquired. Special hospitals and cottage hospitals will, no doubt, be made to contribute to the general training of nurses through some scheme of affiliation with large general hospitals. The partially-trained and unregistered nurse will certainly no longer be able to claim the same fees as her fully-trained and registered sister, but this can hardly be considered an injustice, seeing that the former has spent neither time nor money in acquiring a training, and can therefore afford to work for a lower remuneration than can those who have invested both in equipping themselves for their profession.

### THE BATHING INCIDENT.

"The Medical Superintendent" of St. Mary (Islington) Infirmary, Highgate Hill, N., writes: My attention has been drawn to a Note in The Hospital of October 8 on "The Bathing Difficulty," from which it appears that the death of an aged woman was accelerated by a bath. This institution is referred to, but nothing of the kind has occurred here.

[St. Mary (Islington) Infirmary was not mentioned in the Note. The incident occurred in the infirmary, or infirm ward, in the Islington Workhouse. The two institutions are, of course, quite distinct.—Ed. The Hospital.]

### GOOD NURSING.

Sister Grace writes: It seems strange that the interesting point raised by "Night Superintendent" in The Hospital of October 1st concerning the best way of preparing patients for operation with regard to the giving of purgatives and enemas has not induced other nurses to give their views on the subject. It has always seemed to me a vitally important matter and one that hardly receives adequate attention from doctors. They usually, and rightly so, leave the nurse in charge to attend to a matter which certainly belongs to her department, but it would be well sometimes if they made due inquiries as to how the necessary arrangements had been carried out. It puts a nurse on her mettle, and if she is in any way deficient in training, common sense, or observation it may obviate difficulties. I entirely agree with "Night Superintendent" that many patients are utterly worn out and exhausted from a disturbed and wretched night, when they are put on the operating table, and all because common sense has not been brought to bear. Unfortunately in hospital it very often happens that the surgeon only announces in the afternoon or evening of the day prior to the operation that it is his intention to take the case the next day. Under those circumstances there is no room left for management; but if time permits it is best to do as "Night Superintendent" suggests and have the matter over, so that the patient has a chance at least of an undisturbed and peaceful night. Perhaps some other nurses will give their views on what is a really important point.

NURSING IN A COUNTRY INFIRMARY.

"M. E." writes:—There are many difficulties to be encountered in nursing in a small infirmary which are never thought of in a large institution. For the past six months I have been superintendent nurse in a small workhouse infirmary in the country, and think that perhaps some of my experiences may interest my sister nurses. Leaving a large training school, where for some years I had been sister, I took up my present position. The infirmary is very pleasantly situated in a beautiful country, is newly built, and quite up to date; the wards are bright and fresh, and there are pleasant lawns for the patients to take exercise. The nurses' quarters, which are at the top of the infirmary, are comfortable, and entirely separate from the patients'. Walking casually through one would say what a delightful place in which to live and work. The staff consists of two assistant nurses, one for day and one for night duty, with myself, and, of course, women from the workhouse to do the cleaning. The most serious difficulty which presents itself is, first, how to get and then how to keep suitable nurses. We advertise and receive replies more or less unsuitable, and when a candidate is elected she may or may not fulfil her part of the contract by coming on the appointed day; or, if she does come, the loneliness and want of excitement in the work more than counterbalance the beauty of the surroundings—for most of our cases are infirm and chronic, with an occasional acute case and a few maternity cases at intervals—then she sends in her resignation, and we begin again to look for a nurse. From the foregoing it will be seen that under those circumstances a superintendent nurse's life may be hard without the heavy cases and worry of a sister in a large hospital or infirmary. I often wonder what remedy could be applied for such a state of things, for it is bad for both patients and hospital when nurses change so frequently, and most disheartening for anyone in charge.

### Leicester Nurses and the Pension Fund.

On Monday last week Mr. Louis H. M. Dick, secretary of the Royal National Pension Fund for Nurses, addressed a meeting of nurses on the aims and objects of the Fund at the Trained Nurses' Institution, Aylestone Road, Leicester. Amongst those present were Miss Rogers, the matron of the infirmary, Miss Pell-Smith, and about 40 nurses. After the meeting the nurses were entertained at tea by Miss McHardy, the lady superintendent of the Nurses' Institute.

### TRAVEL NOTES AND QUERIES.

By our Travel Correspondent.

The Riviera on Five Francs a Day (Broomlea).—You give no pseudonym, but I hope you will see this. I do not think it possible for you to find accommodation at terms so low as 5 fcs. per day except in very out-of-the-way spots, which would be entirely unsuited to a person who ought to have bright surroundings. There is a convalescent home at Mentone which would exactly suit you, should you be fortunate enough to gain admission. It is called the Villa Helvetia. Terms 20s. per week. Send stamped and addressed envelope to Messrs. Barclay, Bevan and Company, bankers, London, asking for rules and conditions of admission. I do not remember their address, but a Directory will give it you. The next best thing I can suggest is the Pension Santa Maria at Mentone, where, for a long stay, I think you might be taken for 6 fcs. or 6½ fcs. a day if you had a north room. This is naturally a disadvantage, but as you are well enough to be much out of doors it would not be of serious consequence.

### Appointments.

Battersea Branch of the Clapham Maternity Home.—Miss Rose F. Grylls has been appointed matron. She was trained at the Cornelia Hospital, Poole, and the Great Northern Central Hospital, Holloway. For the past three years she has been sister at the Clapham Maternity Hospital. She holds the L.O.S. certificate.

Birkenhead Borough Hospital.—Miss Edith M. Moore has been appointed sister. She was trained at the Birkenhead Union Infirmary, where she has since been sister.

Bolton Borough Hospital for Infectious Diseases.—Miss Marian Stevenson has been appointed matron. She was trained at Dundee Royal Infirmary and Belvedere Fever Hospital, Glasgow. She has since been assistant matron at Belvedere Fever Hospital, Glasgow.

Burton-on-Trent General Infirmary.—Miss Beatrice Smith has been appointed sister. She was trained at the North Riding Infirmary, Middlesbrough, and has since been staff nurse at the Derbyshire Royal Infirmary.

County Council of Salop.—Miss Ethel L. Frith has been appointed inspector of midwives and lecturer on hygiene, etc., for the county of Salop. She was trained at the Staffordshire General Infirmary, received district training at Cardiff, and midwifery training at Gloucester. She has been Queen's nurse at Gainford, Durham, and holds the L.O.S. certificate.

Gloucester Isolation Hospital.—Miss Elizabeth Yeats has been appointed matron. She was trained at the Nightingale School in connection with St. Thomas's Hospital and at the London Hospital. She has since been matron of Gloucester General Infirmary and of the Gloucestershire Eye Institution.

Harrogate and Knaresborough Joint Hospital.—Miss Emily Morgan has been appointed matron. She was trained at the Royal Infirmary, Newcastle-on-Tyne, where she was afterwards charge nurse of wards and acting night superintendent and theatre sister. She has since been matron-nurse of the Knight Memorial, Blyth, matron of the Wallsend Joint Hospital, and matron of the Crewe Borough Hospital. She holds the Glasgow diploma for midwifery.

Hospital for Women and Children, Leeds.—Miss Letitia M. Hayman has been appointed sister. She was trained at the Middlesex Hospital, and has since done private nursing at the Blackheath Nursing Institute, and been staff-nurse at the Royal Bath Hospital, Harrogate.

Johnstone Combination Hospital for Infectious Diseases.—Miss Isobel Rylands has been appointed matron. She was trained at Crumpsall Infirmary, Manchester, and for the last three years she has been matron of the Victoria Hospital, Dunoon.

Nottingham Children's Hospital.—Miss Allsop, Miss Stericker, and Miss Cook have been appointed sisters. Miss Allsop and Miss Stericker were trained at St. Bartholomew's Hospital, London, and Miss Cook at Addenbrooke's Hospital, Cambridge.

St. Luke's Workhouse Infirmary, Shepherdess Walk, City Road, London.—Miss Grace Dean has been appointed assistant nurse. She was trained at St. Joseph's Hospital, Burlington Lane, Chiswick, by the Meath Workhouse Nursing Association.

Stirling District Asylum, Larbert, N.B.—Miss Christina Fraser has been appointed assistant matron. She was trained at the Western Infirmary, Glasgow, where she has since been holiday sister.

Tredegar Park Cottage Hospital, Tredegar.—Miss Annie Bramwell has been appointed matron. She was trained at Charing Cross Hospital and the City of London

Lying-in Hospital, and has since been charge nurse at the Park Fever Hospital, London, night sister at the Shrewsbury Infirmary, matron of Llandrindod Wells Hospital, and matron of Axminster Cottage Hospital.

Tynemouth Union.—Miss Edith Annie Maud Taylor has been appointed charge nurse. She was trained at Carlisle Union Hospital, and has since been private nurse and monthly nurse at Hull.

Whitehaven and West Cumberland Infirmary.—Miss Lydia Ritson has been appointed sister. She was trained at the Chesterfield and North Derbyshire Hospital and has since been staff-nurse at the Royal Infirmary, Sheffield, and the Kendal Hospital, theatre-sister at the Hartlepools Hospital, and charge-nurse at the Fever Hospital, Stockton-on-Tees. She has also done private nursing.

Workhouse Infirmary, Caterham Union.—Miss Charlotte Dixon has been appointed charge nurse. She was trained at Woolwich Union and Garrison Women's Hospital, and has since been nurse at Greenwich Union and senior nurse at Whitehaven Union.

### Novelties for Nurses.

By Our Shopping Correspondent.

### EGERTON BURNETT'S DRESS MATERIALS.

From Egerton Burnett's Royal Serge Warehouse, Wellington, Somerset, I have received a large collection of patterns of autumn and winter dress materials. Besides innumerable varieties of the famous serges, a wide range of tweeds, hopsacks, merinos, cashmeres, many fancy serges and other woollen materials are included. A particular set of patterns of nurses' dress materials reminds me that Egerton Burnett makes a speciality of woollens and cottons for nurses. These include a large selection of thoroughly reliable fabrics for uniforms, such as galateas, zephyrs, drills, etc., from 7½d. a yard, and I would strongly recommend these washing materials. There are also navy-blue, grey, and black dress serges from 1s. 11½d. a yard, and other woollen dress fabrics from 1s. 3½d. Waterproof cloaking serges are equally moderate in price. Special quotations are given to hospitals and nursing institutions for quantities of any kind of material of 50 yards and upwards. All serges, tweeds, etc., can be made up in Egerton Burnett's tailoring department for a very moderate charge; nurses' cloaks, too, can be made to measure in any shape, and for either costumes or cloaks there are also some delightful alpacas. Then there is an excellent white apron linen, 45 inches wide, at 2s. a yard. Nurses' pure linen cuffs and collars can also be obtained from the warehouse. Of white and red flannels some 50 patterns have been sent; and it would be difficult indeed not to make a satisfactory selection from such a variety. I would draw the attention of nurses to a beautiful novel fabric of pure wool called "Kosmousinos," for dressing gowns and underclothing, which is specially recommended to persons suffering from sciatica, gout, or rheumatism. If those readers who are renovating their wardrobes will send for Egerton Burnett's special nurses' price list, and patterns of materials, they will, I feel sure, find the very thing they need. And any who are thinking of off-duty garments, particularly fancy blouses, or evening dresses, should ask to see patterns of an ideal make of crêpe de chine, in black and many beautiful colours; also a nice soft silk called "Fashionable Silk" in white, and some very delicate and becoming shades of pale blue, pink, green, and mauve.

### "DOMEN" BELTS AND CORSETS.

"Domen" belts and corsets have for some time past had an acknowledged place in a front rank where moderate prices, simplicity, and efficacy are valued. And to the already large variety of "Domen" appliances constant fresh designs and improvements are being added. When I recently called upon the manageress at the chief depôt she showed me several new productions. The well-known "Domen" belt-corset is now also made in a very low bust style, and to wear with this the manufacturers have just brought out a useful "Bust-Girdle," made in white washing material. Nurses and others who have already found comfort from wearing a "Domen" belt-corset will welcome this new shape designed to meet the present fashions. I was also shown a very nice corset without any belt, called the "Graceease." This corset, as is the case with all "Domen" corsets, is fitted with narrow elastic insertion each side, to give freedom to the respiratory organs. A special "Domen" construction for displacement of the kidneys is a pad made to be used inside a belt-corset, thus avoiding the double thickness inevitable when corset and belt are worn as two garments. Of belts there are a very large variety of every size and shape for men, women, and children, all of which seem most carefully and skillfully designed. The manageress drew my attention to two new makes of belts. One, a hypogastric belt for corpulency and general support, is fitted with a peculiarly constructed flexible steel spring with a well-cushioned pad which maintains an equable and gentle pressure on the lower part of the abdomen. The other was a neat-looking shallow belt-bandage in grey elastic, useful where a deeper belt is unnecessary. All the "Domen" corsets and belts are also made in white canvas for summer wear. The Company makes an excellent "stoop cure," which looks a very prompt and efficient corrective to stooping. All the productions are stocked in a great variety of sizes and materials, while special requirements can be made to order, and any particular design carried out. The manageress herself fits ladies at the Strand depôt, and fitters are also sent out to ladies' houses and to the hospitals. The address of the chief depôt of the "Domen" Belt Company is 456 Strand, London, W.C.

### A NURSE'S CLOAK.

Messrs. Matthew Rose and Sons, of Mare Street and Amhurst Road, Hackney, London, N.E., have submitted for approval a nurse's cloak which can only be described as of exceptional value, since the price is but 25s. 9d., and the material, cut, and finish excellent. This cloak, which is made in black and navy blue, has a beautifully shaped deep cape with a detachable velvet collar. Every detail in the make of the garment is admirable, and evidently the work of a very good tailor. The cloak is fastened with large smoked pearl buttons and thoroughly good button-holes; the cape also buttons all the way down in front, thus preventing that fly-away look often given by a half-fastened cape. Altogether it would be impossible to find a neater, smarter, or more sensible-looking cloak, and it is certainly one that can be most confidently recommended. The cloak is a special line of Messrs. Matthew Rose and Sons, and is worth considerably more than 25s. 9d.

### COTTON-WOOL UNDERCLOTHING.

I have received patterns of Dr. Lahmann's cotton-wool fabrics for underclothing, with a new illustrated price list of the garments into which they are made. The materials are of a stockingette nature, woven in several textures, and are either natural coloured or bleached white. People with sensitive skins who cannot tolerate animal wool but who wish for a warm, or cool, material that is porous and elastic, should find this underclothing valuable; and, besides having a very soft finish, it possesses the further advantages of being unshrinkable, durable, and even mothproof. Although the name suggests that the material might be somewhat inflammable, I have proved that when on fire it burns slowly and not at all after the ominous, flaring manner of flannelette. Every kind of undergarment for men, women, and children is made in the "Reform Cotton-wool"; and children's underclothing, including prettily-fashioned white layettes, is a speciality. Dr. Lahmann is the proprietor and resident head physician of the well-known Lahmann Sanatorium, Auf Weisser Hirsch, near Dresden; and the London wholesale and retail agency for the underclothing is at 15E Fore Street, E.C.

### A NOVELTY IN CORSETS.

The Knitted Corset Company, of Mansfield Road, Nottingham, make a specialty of corsets of all kinds. Their original knitted corset is made of a knitted mesh, with the usual whalebone supports. This renders the corset elastic, warm, and shapely. The knitted mesh can be of cotton, wool or silk as required. But the company supply corsets of all kinds besides, and will make to order from any pattern supplied. Their latest novelty is the Hercules unbreakable steels to supersede whalebone. They are very elastic, strong, light, and do not rust. They spring back into shape much more readily than whalebone, so that the shape of the corset is maintained much longer. A sample steel is issued with the catalogue, which is very fully illustrated and an excellent guide to purchasers.

### BUTTER SCOTCH.

Callard and Bowser's butter scotch is too well known to need description, and the convenient packets sold by every confectioner are familiar to all. Now the makers are supplying this delicious sweetmeat in tasteful boxes, which will, we think, be very popular. A present of butter scotch is always welcome, and if daintily encased it is more suitable and attractive for presentation.

### A NIGHTGOWN FOR INVALIDS.

Both nurses and patients know how inconvenient the ordinary form of nightgown proves in illness. Very often a garment is sacrificed to facilitate matters, and the scissors are ruthlessly applied. Messrs. Cash, of Coventry, can now supply an invalid's nightdress, which renders discomfort or demolition unnecessary in the future. Well-concealed buttons and button-holes, enable each limb to be uncovered separately and the toilette of the patient to be completed without the usual fatigue of taking off and putting on of a garment. Besides the fastenings being imperceptible in like manner it can be entirely removed with ease; the nightgown is prettily fashioned. It is supplied in various sizes and materials, and doubtless Messrs. Cash will be glad to send all particulars to inquirers. The design is by Mrs. Francis, and it has, we understand, been registered.

### New Books on Nursing.

Outlines of Routine in District Nursing. Drawn up for the use of District Probationers. By M. Loane, Superintendent of District Nurses, Portsmouth. (Price 8d.)

The Incidental Opportunities of District Nursing. By M. Loane. (Price 3d.)

The District Nurse as Health Missioner. By M. Loane and H. Bowers, Health Visitor, Nottingham. (Price 3d.)

District nursing is much to the fore nowadays, and we welcome anything which is likely to help forward the movement and raise the standard of such an important branch of nursing, touching so closely as it does the welfare of our national life. These pamphlets have been written with this purpose in view, and should be most useful to district nurses and all who are interested in work among the poor. Miss Loane writes from well-proved experience and many years of observation. Her "Outlines of Routine" should prove very valuable in the hands of a district probationer who, though she may be a three-year certificated nurse straight from hospital, may well feel at a loss how to manage in a poor cottage, where all the things she has been accustomed to have at hand are conspicuous by their absence. By adopting the suggested methods of routine, the nurse will soon learn to "make her head save her heels" and get through her round more quickly and with the minimum of fatigue to herself and trouble to the patients' friends. The lessons are well thought out, clear, and concise, though an unnecessary amount of detail is occasionally introduced. For example, in Lesson V. a list of things to get ready to place on the clean newspaper has been given—it might be left to the nurse's common sense where to place them—"right hand near corner," "right hand far corner," "right of centre," "left of centre," etc., is almost puzzling! The other two pamphlets bring into strong relief the higher aims of district nursing, and point out the many opportunities the nurse has for doing good in various ways. Not until the nurse fully grasps this wider aspect of her work will she become a truly successful district nurse, a health missioner, and a power for good. The pages contain a great deal of instruction and much practical advice. We recommend their perusal to all district nurses.

A Manual of Fever Nursing. By Reynold Webb Wilcox, M.D. (London: Rebman, 1904. Pp. 236. Price $1.00 net.)

The first part of this volume is occupied with a description of fever in general. The symptoms usually associated with it are discussed one by one, and a clear and accurate account given of the appropriate treatment. The duties of the nurse are detailed at some length, together with many useful hints on such subjects as taking the temperature and counting the pulse and respiration; and special attention is devoted to the diet and the methods employed to reduce the temperature. There is a useful chapter on the preparation and management of the sick-room, and the precautions to be observed in infectious diseases. The whole of this part of the subject has been very fully treated, and a nurse will find all that it is necessary for her to know. In the second half of the book brief individual descriptions are given of many of the febrile diseases, including some such as yellow fever, mountain fever and dengue, which are not met with in this country. For the most part the accounts are clear and sufficiently complete, but in places there are omissions which somewhat detract from the value of the book. Especially is this the case in dealing with the nursing of the infectious diseases. For instance, in the section on diphtheria, no account is given of the treatment of heart failure and paralysis, or of the treatment after tracheotomy, although room has been found for a full description of the method of taking bacteriological cultivations from the throat. There is no mention of croup among the complications of measles, and no reference is made to the peculiar delirium of small-pox and the importance of watchfulness on the part of the nurse. Probably these omissions are due to considerations of space; but we cannot help thinking that the book would have been of more value to English nurses if fewer diseases had been described, and if the special nursing of each had been given in fuller detail. Some curious inaccuracies are met with here and there. Thus the incubation period of scarlet fever is said to be one week usually, and the process of desquamation is stated to continue from two to three weeks. Rheumatic nodules and erythema nodosum are described as identical. The book is well arranged and clearly written, and contains a number of useful temperature charts illustrative of various diseases.

### "The Hospital" Convalescent Fund.

The Hon. Sec. acknowledges with many thanks the receipt of 2s. 6d., per the Travel Correspondent.

### Echoes from the Outside World.

### Movements of Royalty.

The King concluded his stay at Balmoral on Sunday, and arrived at Euston on Monday night. The Royal train did not stop between Carlisle and London, and accomplished the distance—300 miles—within six hours and a quarter. On its arrival his Majesty shook hands with the superintendent of the line, and thanked him for the admirable arrangements which had been made for the journey. A large crowd had assembled outside the station, and the King received a loyal and cordial welcome back to London. The Queen, who concludes her stay in Denmark this week, paid a visit of condolence to Madame Finsen last week.

### Lady Curzon's Illness.

Following a serious relapse at the end of last week the condition of Lady Curzon again improved, and on Tuesday it was announced that she continued to make slow, but gradual, progress. It was, however, stated in the bulletin that the complication of phlebitis had supervened. Another relapse occurred on Tuesday, and on Wednesday the report was that the condition of the patient remained serious.

### The War Between Russia and Japan.

Lord Lansdowne, in reply to a suggestion of the Committee of the International Arbitration and Peace Association that his Majesty's Government should, in concert with the other Powers, appeal to the Governments of Russia and Japan to desist from hostilities, states that neither of the belligerents having expressed any desire for mediation, his Majesty's Government do not consider that they could with advantage take such action. In an order of the day issued by General Kuropatkin to his troops on October 2nd, he says that the Manchurian army has not hitherto been numerically strong enough to defeat the Japanese. It was for this reason that, in spite of the repeated repulse of Japanese attacks on the Russian positions he gave the order to retreat, with a sorrowful heart, but with unshaken confidence, that it was necessary in order to gain a decisive victory when the time came. He affirms, however, that the Emperor has assigned for the conflict with Japan, forces sufficient to assure victory to Russia; that all the great difficulties of transport are being overcome; and that if the regiments already sent out prove insufficient, fresh troops will arrive, "for the inflexible wish of the Emperor that the foe should be vanquished will be inflexibly fulfilled." The occupation of Ben-tsia-putse by the Russian troops is officially confirmed, and the abandonment of the place by the Japanese without a struggle is interpreted as indicating their intention to concentrate all their strength in the defence of Liau-yang. The Mikado has issued an appeal to his people urging patience and steadfastness in the pursuance of the war.

### The Church Congress and Religion in the Home.

At the concluding meetings of the Church Congress at Liverpool several ladies were among the speakers. After Colonel Seton-Churchill had declared that when he looked at some of the empty-headed society girls of the present day he frequently trembled for the future mothers of England, and that it was perfectly sickening to see the weary round of frivolity and so-called pleasure going on in some houses, where Sunday was turned into a kind of weekly Bank Holiday, Lady Frederick Cavendish strongly denounced the fashionable "week-end" which so often tended to the subversion of the Fourth Commandment, which was the foundation-stone of Sunday in general and in the home in particular. Mrs. Sumner said that the fact that many children of the educated classes were not instructed by their parents in the knowledge of the Bible and the creed of the Christian, and that the Bible was passing out of our national education in a great measure, was dealing a deadly blow at religion and at character. Canon McCormick dwelt upon the necessity of checking in the home the craving for outside amusements and recreation on the part of the younger members of the household by making home the most attractive place in the whole world.

### Death of Isabella Bishop.

The death of Mrs. Isabella Bishop, traveller and author, occurred at Edinburgh, on Friday. She was in her 72nd year, and had been ill for some time. Lately, however, she rallied, and was removed a little time ago from a nursing home to a residence in Melville Street. As Miss Bird, the deceased lady began to travel on account of ill-health, when she was only 22, thirty years before her marriage to the late Dr. John Bishop. She had probably seen more of the world at large, and of Asia in particular, than any of her female contemporaries. She did not travel only for pleasure or information, but greatly interested herself in medical missions, and no fewer than five hospitals and an orphanage in the East were established in consequence of her untiring exertions. In 1892, after five years' travel in Asia, she was made the first lady Fellow of the Royal Geographical Society. The names of her books constitute perhaps the best record of her many journeys. Commencing with "The Englishwoman in America," in 1851, she subsequently published "Six Months in the Sandwich Islands." "A Lady's Life in the Rocky Mountains," "Unbeaten Tracks in Japan," "The Golden Chersonese," "Journeys in Persia and Kurdestan," "Among the Tibetans," "Korea and her Neighbours," "The Yangtze Valley and Beyond," and "Pictures from China." Mrs. Bishop was a clever photographer, and an enthusiastic botanist, and from her childhood she studied chemistry, and the use of the microscope. She was accustomed to say that in all her travels her sex was really a protection rather than a danger, and it was not until she travelled in China that she carried any arms at all.

### Mr. Carnegie's Latest Gift.

It was announced at a meeting of the Islington Borough Council on Friday evening that Mr. Andrew Carnegie had offered the sum of £40,000 for the purpose of forming in Islington a central library building and two branch libraries, provided that a penny rate for their support were levied without delay. The Council unanimously passed a resolution in favour of accepting the offer, which was characterised as a princely gift.

### New Play at the Imperial Theatre.

Mr. Lewis Waller has again made a fortunate venture at the Imperial Theatre. His new piece, which is called "His Majesty's Servant," is written by two ladies, Sarah Barnwell Elliott and Maud Hosford, and deals with the days of Cromwell and Charles II, a period naturally lending itself to much beauty in dress. The hero, Mohun, is not unknown to history, and was a King's player at the Restoration with the title of Major, but his Christian name then was Michael, not Geoffrey. In the play it is as a soldier that he shines forth, a soldier, moreover, who, in the first act, has the honour of helping Prince Charles to escape from the celebrated oak, and also of rescuing the beautiful Lettice Pierpoint, the heroine. Later, Mohun is the moving spirit in the negotiations with Monk for the return of the King, and of course he is ultimately successful, though, owing to the machinations of the evil Damaris Holden, the King is once nearly betrayed, and Mohun nearly loses his life. Miss Evelyn Millard is the winsome heroine, and Mr. Waller is well suited as the witty, audacious, and brave soldier who is the *deus ex machinâ* of the piece.

### Notes and Queries.

**FOR REGULATIONS SEE PAGE 12.**

*Home for Crippled Children.*

(14) May I trouble you for the address of the Duchess of Sutherland's Home for Crippled Children, or some other home where girls are trained as nurses when too young to enter a general hospital?—*Nurse B.*

The Duchess of Sutherland's Home is at Hartshill, Stoke-on-Trent. With regard to preliminary training, for a full list of institutions see "The Nursing Profession: How and Where to Train."

*Heart Case.*

(15) Will you kindly let me know if a heart case, 84 years of age, having had marked Cheyne Stokes breathing five months ago, will be likely to make a permanent recovery?—*Constant Reader.*

It is quite impossible to give an opinion without seeing the patient.

*Home.*

(16) Do you know of a home or institution that would take me in? I am a retired solicitor, a man of 70 and of some means. I am afflicted with prostatitis and also with frequent attacks of gastritis and liver.—*J. M. A.*

Advertise and you will probably have suitable replies.

I am anxious to place my husband in a home for paralysis. I should not trouble you but feel that you will give me reliable information.—*N. J. B.*

We cannot recommend private homes. A few private patients will be received at the Hospital for Epilepsy and Paralysis, 4 Maida Vale, W., as soon as the new buildings are ready for occupation.

Will you kindly tell me of a holiday home for nurses at Bournemouth? I want to go somewhere for a fortnight where there are shops, amusements in the evening, etc.—*Matron.*

There is no special holiday home for nurses in Bournemouth.

*Dispensing.*

(17) When in hospital I was taught to make up doctor's prescriptions without knowing more of the nature of drugs than a well-trained nurse usually does. Would this knowledge be enough to qualify me to answer advertisements for "nurses to dispense," and if not what qualification should I require?—*B. E.*

You require the minor qualification of the Pharmaceutical Society, 17 Bloomsbury Square. Write for particulars to the Secretary.

*Hospital Training.*

(18) Can you advise me how to get into a hospital for infectious diseases as probationer?—*J. F.*

Write to the lady superintendent of any hospital for infectious diseases. For list see "The Nursing Profession: How and Where to Train." If you wish to be near London those of the Metropolitan Asylums Board would be suitable.

I am a certificated nurse and wish to become a Queen's nurse. Will you tell me where I can obtain the necessary training?—*X. Y.*

Apply the General Superintendent, Queen Victoria's Jubilee Institute for nurses, 120 Victoria Street, S.W.

*Abroad.*

(19) I am informed that there is a good demand for maternity nurses in the South of France during the season. I should be glad if you will inform me if this is correct, and say if it is advisable for a private nurse to begin work on her own account or to join a nursing home?—*E. T.*

A private nurse without introductions or connections would do better to join a nursing home. The Nice Nursing Institute, Villa Pilatte, Avenue Désambrois, Nice, might be able to employ you.

**Handbooks for Nurses.**

"The Nurses' Pronouncing Dictionary." 2s. post free.

"Surgical Ward Work and Nursing." By Dr. A. Miles. Price 3s. 6d. net; 3s. 10d. post free.

"The Human Body: Practical Physiology and Hygiene." 5s. post free.

"The Nursing Profession: How and Where to Train." 2s. net; 2s. 4d. post free.

"Preparation for Operation in Private Houses." Price 6d. post free.

The above may be obtained of all booksellers or of The Scientific Press, Limited, 28 & 29 Southampton Street, Strand, London, W.C.

### For Reading to the Sick.

"LEAD ME, O FATHER."

Lead me, O Father, holding by Thy hand;
I ask not whither, for it must be on. . . .
. . . .
I'll think of Jesus, who hath led my soul
Thus far upon its journey home to God.
By poor attempts to do the things He said,
Faith has been born; free-will become a fact;
And love grown strong to enter into His,
And know the Spirit that inhabits there.
One day His truth will spring to life in me,
And make me free, as God says—"I am free."
When I am like Him, then my soul will dawn
With the full glory of the God revealed—
Full as to me, though but one beam from Him—
The light will shine, for I shall comprehend it:
In His light I shall see light. God can speak,
Yea, *will* speak to me then, and I shall hear.

*George Macdonald.*

We touch and scrutinise the fresh green budding plant, instinct with exuberant life; but everywhere its life evades equally the grasp of the hand, or the perception of the mind. We stand before the living form amazed, perplexed; we pass away, musing, wondering. The same law of the secrecy of life prevails everywhere around us. Probably one great part of the mysterious thrilling joy of the Future will be to look upon the inner workings and seats of life with an understanding heart. This insight, this perception of the presence of life, is not yet given to us.

If, as we offer the acceptable service of a life of sacrifice in Christ, we fulfil the desire of His soul, and are in Him Who is well pleasing to the Father, and thus share His acceptableness, the result must be an unspeakably blessed and glorious reconciliation of mutual rest and delight in a united life and common acts of all-absorbing fellowship and joy unspeakable. More and more, in an ever-deepening truth, we thus "in Him live and move and have our being." For He, and all that He has won for us, becomes ours. His joy in the Father is ours. The Father's joy in Him is ours. It is a unity of life; and all that makes life precious, and all that pleases in the mutual interchange of living powers of love and joy, between the Father and the Son, are ours, who are in the Son, and through the Son are in the Father also.

The only change is that our nature is pervaded by a life and love beyond it, transforming it into a diviner order ever more and more perfectly. And as our efforts prevail to preserve a life of stillness and repose, of faith and love, of prayer and watchfulness, and a pure intention, this diviner life in us is increasingly strengthened and enlarged. All is transformed and raised as more and more we unite ourselves with the amazing mystery of the Presence which is inhabiting our being, working out Its purposes in us, and which is already ours in Its immeasurable and inexhaustible depth of love.—*T. T. Carter.*

Thy pierced Hand guides the mysterious wheel,
Thy thorn-crowned brow now wears the crown of power,
And when the dark enigma presseth sore
Thy patient voice saith "Watch with Me one hour."
As sinks the moaning river in the sea
In silver peace, so sinks my soul in Thee.

## Figures and Tables

**Figure f1:**
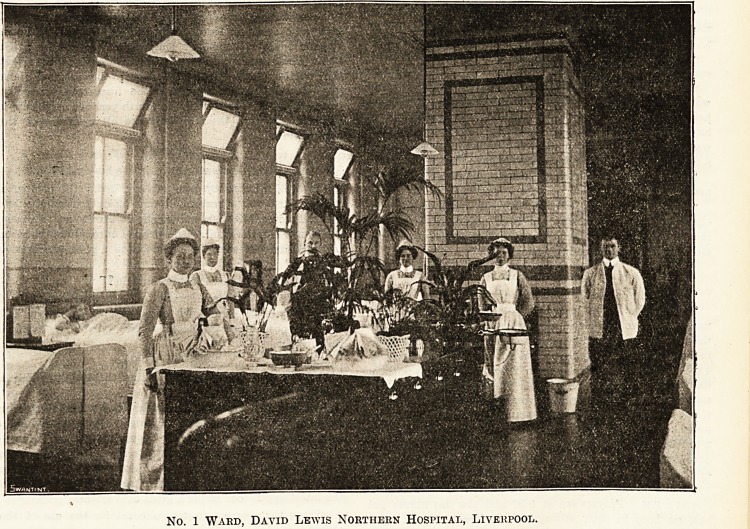


**Figure f2:**
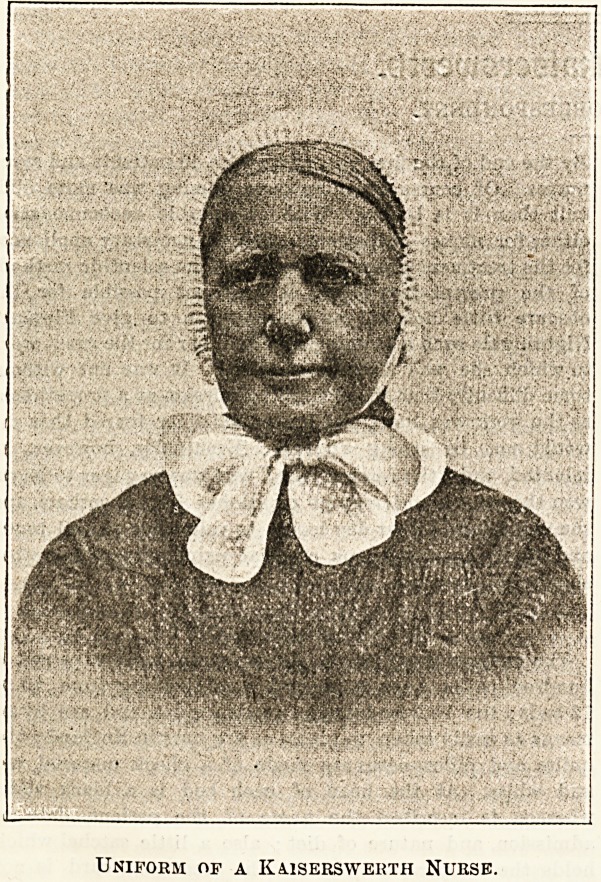


**Figure f3:**
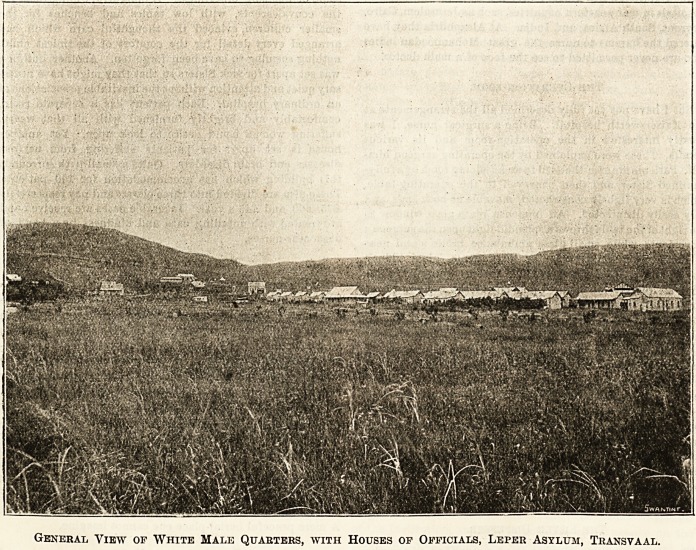


**Figure f4:**